# Potent antitumor effects of cell-penetrating peptides targeting STAT3 axis

**DOI:** 10.1172/jci.insight.136176

**Published:** 2021-01-25

**Authors:** Maryam Aftabizadeh, Yi-Jia Li, Qianqian Zhao, Chunyan Zhang, Nigus Ambaye, Jieun Song, Toshikage Nagao, Christoph Lahtz, Marwan Fakih, David K. Ann, Hua Yu, Andreas Herrmann

**Affiliations:** 1 Department of Immuno-Oncology and; 2 Irell & Manella Graduate School of Biological Sciences, Beckman Research Institute at City of Hope Comprehensive Cancer Center, Duarte, California, USA.; 3 Sorrento Therapeutics, San Diego, California, USA.; 4 Department of Medical Oncology and Therapeutics and; 5 Diabetes & Metabolism Research Institute, Beckman Research Institute at City of Hope Comprehensive Cancer Center, Duarte, California, USA.

**Keywords:** Oncology, Therapeutics, Peptides

## Abstract

To date, there are no inhibitors that directly and specifically target activated STAT3 and c-Myc in the clinic. Although peptide-based inhibitors can selectively block activated targets, their clinical usage is limited because of low cell penetration and/or serum stability. Here, we generated cell-penetrating acetylated (acet.) STAT3, c-Myc, and Gp130 targeting peptides by attaching phosphorothioated (PS) polymer backbone to peptides. The cell-penetrating peptides efficiently penetrated cells and inhibited activation of the intended targets and their downstream genes. Locally or systemically treating tumor-bearing mice with PS-acet.-STAT3 peptide at low concentrations effectively blocked STAT3 in vivo, resulting in significant antitumor effects in 2 human xenograft models. Moreover, PS-acet.-STAT3 peptide penetrated and activated splenic CD8^+^ T cells in vitro. Treating immune-competent mice bearing mouse melanoma with PS-acet.-STAT3 peptide inhibited STAT3 in tumor-infiltrating T cells, downregulating tumor-infiltrating CD4^+^ T regulatory cells while activating CD8^+^ T effector cells. Similarly, systemic injections of the cell-penetrating c-Myc and Gp130 peptides prevented pancreatic tumor growth and induced antitumor immune responses. Taken together, we have developed therapeutic peptides that effectively and specifically block challenging cancer targets, resulting in antitumor effects through both direct tumor cell killing and indirectly through antitumor immune responses.

## Introduction

Signal transducer and activator of transcription 3 (STAT3) is a cytoplasmic signaling molecule and nuclear transcription factor that regulates the expression of diverse genes (1, 2). STAT3 activity is tightly regulated in nonmalignant cells during normal physiology, whereas it is persistently activated in almost 70% of hematological and solid cancers (3). Persistent activation of STAT3 results in deregulation of gene expression, promoting cell proliferation and survival, tumor angiogenesis, and metastasis (4, 5). Furthermore, STAT3 activation in tumors can be transmitted to immune cells in the tumor microenvironment through secreting mediators such as IL-6 among many other cytokines and growth factors. Activated STAT3 in immune cells in the tumor microenvironment in turn induces immune suppression by augmenting production of immunosuppressive cytokines and factors while inhibiting Th1 immunostimulatory mediators, leading to inhibition of antitumor T cell activity (6). Additionally, cytokines and growth factors produced by tumor-associated immune cells and fibroblasts activate STAT3 in tumor cells, forming a feed-forward loop to maintain activated STAT3 in both transformed and untransformed cells in the tumor microenvironment (3, 7, 8).

Although STAT3 as an important target for cancer therapy has been well documented, it is a challenge to develop potent and specific STAT3 inhibitors. Unlike many kinases that usually have well-defined ATP-binding pockets, in which small molecules can be designed to fit and block their functions (9), STAT3 is a transcription factor lacking such pockets. Nevertheless, there are several types of STAT3 inhibitors that are either in clinical testing or used for research purposes.

To date, most of the small-molecule STAT3 inhibitors are designed to target either JAKs or the phospho-tyrosine (pY)-SH2 domain of STAT3, which is critical for STAT3 activation. Many of those compounds remain in preclinical stage due to low efficacy, high off-target effects, or undefined mechanism(s) through which they inhibit STAT3 activity (10, 11). Although some of the inhibitors show promising antitumor effects in preclinical studies and are currently in clinical trials, they can also inhibit STAT1 or STAT5 because of the high similarity of SH2 domains among all members in the STAT family (12, 13). Additionally, several natural products, including curcumin (14, 15) and its derivatives (16), as well as resveratrol (17) or caffeic acid (18), have been shown to inhibit STAT3 activity. Although these natural compounds can decrease STAT3 phosphorylation and/or total STAT3 levels, their exact inhibitory mechanisms are not well defined.

Oligonucleotide-based therapeutics, including siRNA, antisense, or decoy oligonucleotides, have shown promise in targeting STAT3. Oligo-based therapeutics were developed to interfere with DNA binding or transcriptional activity and have, recently, shown improved efficacy in preclinical and clinical trials after modifications to their chemical structure (19, 20). Meanwhile, conjugating CpG motifs, the ligand for Toll-like receptor (TLR9), to oligonucleotides, including siRNA, antisense against STAT3, enables targeted in vivo delivery into TLR9-positive tumor-associated immune cells as well as tumor cells with elevated TLR expression, leading to potent antitumor effects through both antitumor immune responses and direct tumor cell killing (21, 22). However, both siRNA (or shRNA) and antisense-based therapy silences/inhibits STAT3 gene transcripts, without selectively acting on activated STAT3, which may reduce efficacy and/or cause side effects.

Peptide-based therapy has several advantages over small molecules or oligonucleotides as they usually target protein-protein interactions, thereby providing target specificity to selectively block activated targeted molecules. For example, the p53 peptides are designed to disrupt the interactions between p53 and MDM2/MDMX, reducing p53 protein degradation and promoting its tumor-suppressing functions (23). In addition, several MYC peptides can effectively block interactions between MYC and MYC-associated factor X (MAX) necessary for MYC oncogenic properties (24). Thus, high target specificity/selectivity makes peptide-based therapy a promising therapeutic approach to target activated STAT3, as well as molecules that are critical STAT3 upstream activator(s) and downstream effector(s), such as Gp130 and c-Myc. However, therapeutic peptides for clinical applications face stability and cell penetrability challenges. During the last decade, several innovative methods were developed to increase the stability and penetrability of therapeutic peptides, including stapled cyclic (25, 26) and D-peptide (27). These modifications improved the pharmacologic performance of therapeutic peptides. Among them, stapled p53 peptides showed very promising results for T cell lymphoma and some solid tumors in clinical testing (28). However, stapled peptide is usually designed as an α-helix structure to bind its target, which can limit clinical application. Despite the improvement resulting from these innovative modifications, lack of efficient cell penetrability and stability remains a challenging issue for broader and efficacious peptide clinical applications.

Several peptide-based STAT3 inhibitors have also been developed to target STAT3 Tyr705 (Y705) phosphorylation and dimerization (29, 30). However, their biofunctionality can only be reached at very high molarity ratios even in cultured cells (>35 μM) (31, 32), most likely due to poor cell penetrability. Thus, a highly specific inhibitor with effective cell penetrability that targets activated STAT3 is urgently needed. In addition to Y705 phosphorylation of STAT3, reversible acetylation of K685 has been revealed as an additional critical regulator of STAT3 activation (33). We and others have previously reported that K685 acetylation is elevated in breast cancer, colon cancer, and melanoma (34, 35), and the acetylation of STAT3 is critical for its dimerization and activation (33), strongly supporting the concept that acetylated STAT3 is a viable therapeutic target.

In the current study, we sought to create an acetylated STAT3 inhibitor to disrupt STAT3 dimerization and activation while overcoming peptide cell penetrability and stability issues. We have also tested cell-penetrating Gp130 and c-Myc peptides, one of which is critical for STAT3 activation and the other a STAT3 downstream effector, by using our newly developed cell-penetrating antibody in vivo delivery platform (36).

## Results

### Characterization of a cell-penetrating acetylated-STAT3-peptide.

**Figure 1:**
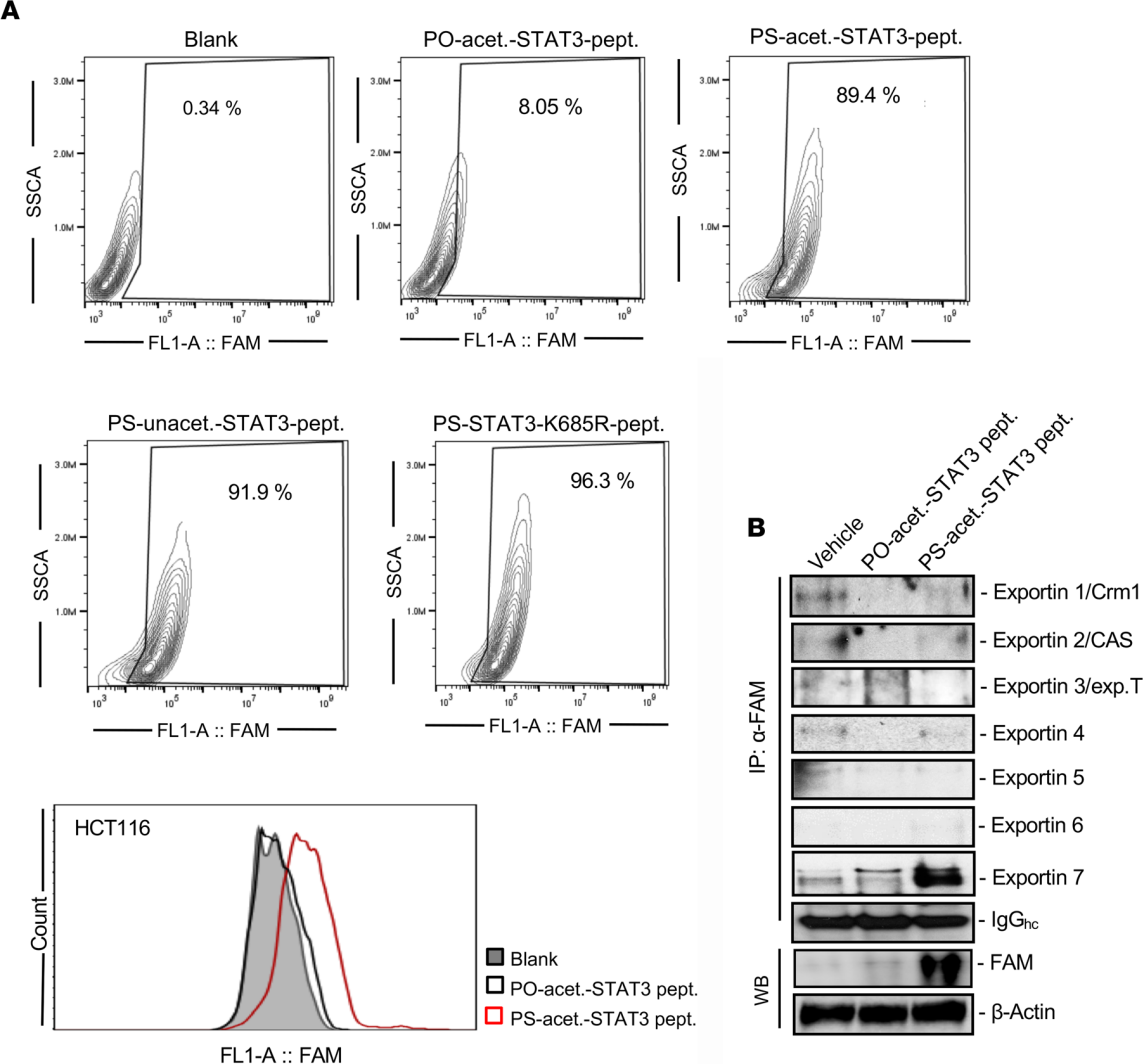
Conjugating PS DNA oligonucleotides enables acet.-STAT3 peptide cell penetration while preserving its specific binding activity to exportin 7. (**A**) The cell penetration efficiency (%) and fluorescence intensity of PS DNA oligonucleotide-modified STAT3 peptide (FAM labeled) in human colon cancer cell line HCT116, as assessed by flow cytometry. Data are representative of 3 independent experiments (*n* = 3). (**B**) Confirmation of the specific interaction between PS-acet.-STAT3 peptide and exportin 7 by immunoprecipitation of the FAM-labeled PS-acet.-STAT3 peptide followed by Western blotting, shown in U251 cells.

To develop a highly selective inhibitor targeting activated STAT3, we designed a 22–amino acid peptide spanning from 675–696 of STAT3 protein (this region of STAT3 is 100% conserved between human and rodent) that includes the K685 acetylation site required for STAT3 activation in tumors (33, 37). Further, we created an acetylated STAT3 peptide by adding an acetyl group to K685 (acet.-STAT3 peptide) (Supplemental Figure 1; supplemental material available online with this article; https://doi.org/10.1172/jci.insight.136176DS1), which is expected to specifically interfere with its protein-protein interaction with exportin 7 and dimerization of acetylated STAT3, leading to subsequently inhibition of STAT3 activation (phosphorylation at Y705). Based our previous results showing that conjugating phosphorothioated (PS) DNA oligonucleotides to antibodies enables their cell penetration (36), we tested whether the same modification can allow effective peptide cell penetration, target binding, and regulation. The acet.-STAT3 peptide was conjugated to either PS-polymer backbone or polymer backbone without phosphorothioated (PO), using a carbon chain linker (Supplemental Figure 1). To further confirm the specific binding and inhibitory effects of PS-acet.-STAT3 peptide, we also generated PS-conjugated STAT3 peptide without acetyl modification (PS-unacet.-STAT3) or PS-conjugated STAT3 peptide with the K685 acetylation site-specific mutation (where lysine is replaced by arginine; PS-STAT3-K685R) (Supplemental Figure 1). To evaluate whether PS modification is required for high efficiency of PS-modified STAT3 peptide penetration into tumor cells, the cell-penetrating efficiency and fluorescence intensities of fluorescein-labeled (FAM-labeled) PS- and PO-modified acet.-STAT3 peptide in human colon cancer HCT116 (Figure 1A) and human glioma U251 (Supplemental Figure 2) cells were measured by flow cytometry after 15 minutes’ incubation under general cell culture conditions. PO-modified peptide was used as a control peptide. The results revealed that, unlike PO-modified peptides, PS-modified peptides were highly efficiently taken up (≥80%) by the cells (Figure 1A and Supplemental Figure 2). Therefore, PS conjugation is essential for high efficiency of intracellular peptide delivery.

Our prior work using the same modification to enable the highly efficient cell penetration of antibody suggests that depolarization of cell membrane contributes to antibody cell entry (36). To test whether alteration in membrane potential also plays a role in internalization of PS-acet.-STAT3 peptide, we induced membrane depolarization with potassium chloride (KCl) in HCT116 cells. Our results indicated that membrane depolarization significantly reduced peptide internalization in the cells (Supplemental Figure 3).

**Figure 2:**
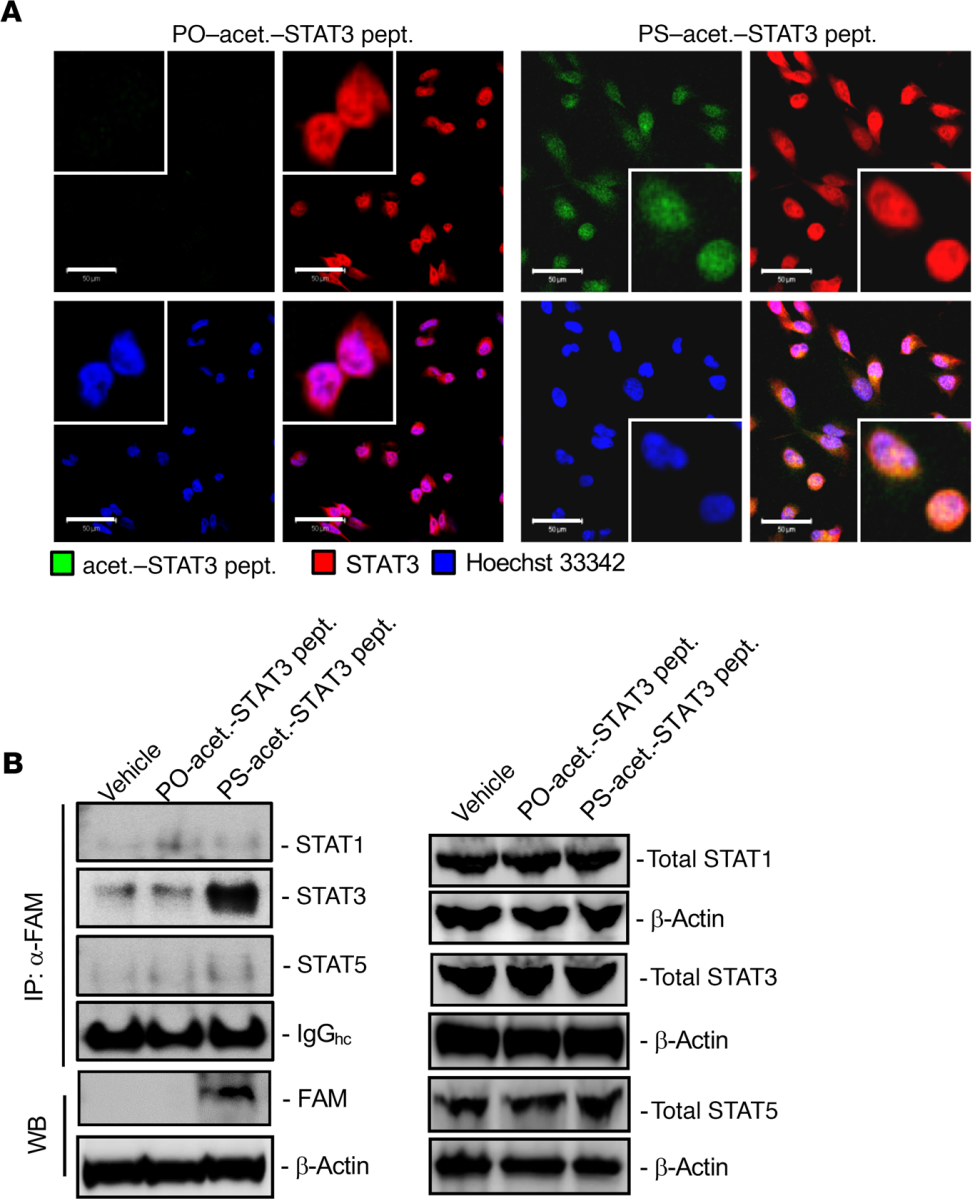
PS-acet.-STAT3-peptide specifically binds STAT3 in the nucleus. (**A**) Penetration of PS-acet.-STAT3 peptide and its colocalization with STAT3 protein in U251 cells are confirmed by confocal microscopy. Scale bars: 50 μm. Insets: original magnification, ×40. (**B**) PS-acet.-STAT3 peptide specifically binds to STAT3 protein, not STAT1 and STAT5 proteins, shown in U251 cells by immunoprecipitation followed by Western blotting (left panel). Expression of total STAT1, STAT3, and STAT5 was confirmed by Western blotting in U251 cells (input protein level, right panel).

In addition to its role in dimerization and DNA binding (33, 34), acetylated STAT3 interacts with exportin 7 at STAT3’s acetylation site (K685) for its nuclear exporting (38). We further investigated whether PS-acet.-STAT3 peptide could interfere with the protein-protein interaction between STAT3 and exportin 7, thereby disrupting STAT3 nuclear exporting. To test to what extent PS-acet.-STAT3 peptide might bind to acetylated STAT3 protein and further disrupt its protein-protein interaction with exportin 7, we performed immunoprecipitation assay with an anti-FITC (FAM) antibody followed by Western blotting. Our results revealed that PS-acet.-STAT3 peptide (FAM-labeled) bound to exportin 7 but not to exportins 1–6 in cells (Figure 1B). Additionally, we confirmed the internalization of FAM-labeled PS-acet.-STAT3 peptide in cells by confocal microscopy. Confocal images of immunofluorescence (IF) staining indicated that the internalized PS-acet.-STAT3 peptide colocalized with STAT3 protein in the human tumor cell line (Figure 2A). To test whether PS-acet.-STAT3 peptide specifically interacts with STAT3, we performed immunoprecipitation, followed by Western blotting. The result showed that PS-acet.-STAT3 peptide specifically bound to STAT3 protein in the cells but not to STAT1 and STAT5 proteins (Figure 2B). We further compared the specificity of PS-acet.-STAT3 peptide with the most advanced clinical small-molecule STAT3 inhibitor, napabucasin (BBI608), currently under several phase III clinical trials (39–41). Napabucasin has been shown to target cancer stem cells through blocking many different pathways, including STAT3 (42, 43). We treated HCT116 tumor cells with either napabucasin or PS-acet.-STAT3 peptide, followed by Western blotting to assess phosphorylated STAT3 (p-STAT3) and p-STAT5 levels. In contrast to napabucasin, which inhibited both p-STAT3 and p-STAT5, PS-acet.-STAT3 reduced only phosphorylation of STAT3 but not of STAT5 (Supplemental Figure 4).

Our prior work with the cell-penetrating antibody suggested a requirement of intracellular target for the retention of PS antibodies (36). We therefore addressed whether the accumulation of PS-acet.-STAT3 peptide in cells requires intracellular acetylated STAT3. To investigate this, both WT and K685R mutant HCT116 cells were treated with FAM-labeled PS-acet.-STAT3 peptide, and the fluorescence intensity of FAM-labeled peptide in cells was measured by flow cytometry. We detected higher fluorescence intensity in the WT cells compared with their K685R mutant counterparts (Supplemental Figure 5A) after peptide treatment. In addition, PS-acet.-STAT3 peptide directly bound to acetyl-STAT3 (Supplemental Figure 5B). Furthermore, we treated HCT116 xenografted tumors with PS-STAT3 peptide without acetylation (PS-unacet.-STAT3), PS- STAT3-K685R (in which lysine 685 is replaced by arginine), and PS-acet.-STAT3 peptides. The cellular retention of PS-acet.-STAT3 peptide in tumors in vivo was assessed by fluorescent IHC staining of tumor tissue sections followed by confocal imaging (Supplemental Figure 5C). Our tissue analysis revealed that, relative to the unacetylated PS-unacet.-STAT3 or PS-STAT3-K685R mutant peptide, PS-acet.-STAT3 peptide was retained in tumors at significantly higher levels. Moreover, PS-acet.-STAT3, but not PS-unacet.-STAT3 or PS-STAT3-K685R peptide, effectively inhibited STAT3 phosphorylation (Supplemental Figure 5D), transcriptional regulation (Supplemental Figure 5E), and tumor progression (proliferation marker Ki-67 and angiogenesis marker CD31; Supplemental Figure 5F). Nevertheless, phosphorylation of STAT3 was greatly inhibited by PS-acet.-STAT3 peptide; acetylation of STAT3 was only moderately suppressed under the same treatment (Supplemental Figure 5G). Hence, the protein stability of STAT3 promoted by acetylation of STAT3 was not affected by PS-acet.-peptide treatment (Supplemental Figure 5H).

### Biological functions of PS-acet.-STAT3 peptide.

**Figure 3:**
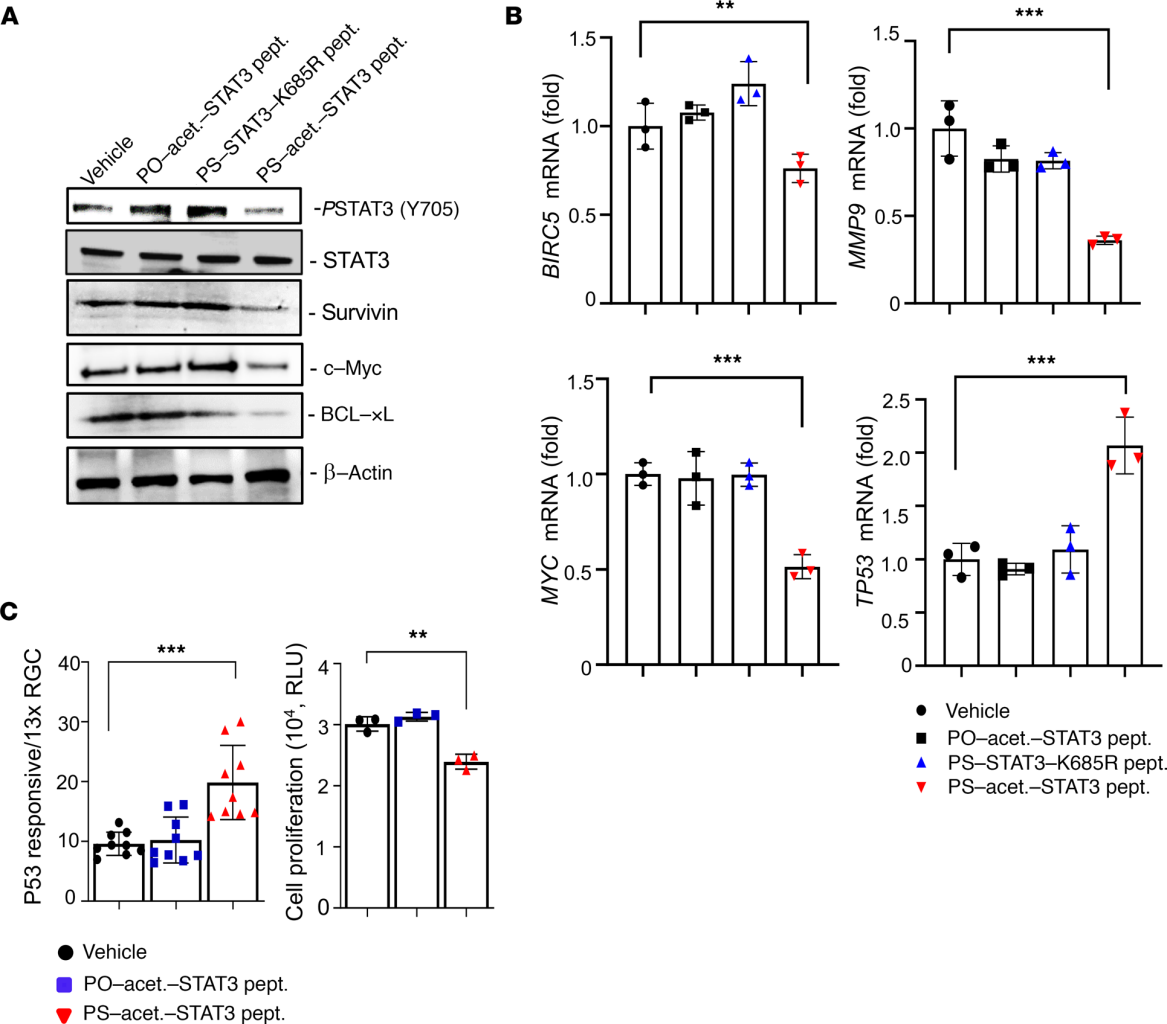
PS-acet.-STAT3 peptide inhibits STAT3 activity and downregulates the expression of STAT3 targets. (**A**) Downregulation of STAT3 activity and its downstream target proteins shown in human HCT116 colorectal cancer cells by Western blotting (data represent 1 of the 3 experiments, *n* = 3). PO-acet.-STAT3 and PS-STAT3-K685R peptides were used in parallel as controls. (**B**) STAT3 inhibition regulated the expression of proliferation and apoptotic genes, assessed by real-time quantitative PCR. Data present mean ± SD, *n* = 3 independent experiments, 1-way ANOVA; ***P < 0.005; **P < 0.01. (**C**) PS-acet.-STAT3 peptide treatment upregulated p53 expression and suppressed cell proliferation in HCT116 colorectal cancer cells. For p53 promoter assay, data represent 3 replicates in 3 independent experiments (*n* = 9). For cell proliferation assay, data represent 3 independent experiments. SD is shown (*n* = 3). One-way ANOVA; ***P < 0.005; **P < 0.01.

We next investigated to what extent PS-acet.-STAT3 peptide diminishes STAT3 activity in tumor cells in vitro. We added PO- or PS-acet.-STAT3 or PS-STAT3-K685R peptides (approximately 1 μM) to the cultured colon cancer HCT116 cells. Western blotting analysis of the cell lysates treated with PS-acet.-STAT3 peptide showed a significant downregulation of STAT3 activation (pY705-STAT3) and the expression of STAT3-regulated genes in protein and mRNA levels, including Survivin (*BIRC5*) and MYC (*MYC*) (33, 34), in HCT116 cell line (Figure 3, A and B). To increase scientific rigor, another colorectal cancer cell line, LoVo, was used to confirm the results from HCT116 cells (Supplemental Figure 6). On the other hand, expression of STAT3-suppressed apoptotic genes, *TP53*, *CASP9*, and *CASP3* (44, 45), were upregulated in cells after PS-acet.-STAT3 peptide treatment (Figure 3B and Supplemental Figure 6). Since STAT3 is known to inhibit p53 expression and tumor cell proliferation (46, 47), we next measured the transcriptional regulation of p53 promoter and tumor cell proliferation in HCT116 cells. Our results showed that overnight incubation with PS-acet.-STAT3 peptide in HCT116 cells led to a significant increase in p53 promoter activity and a decrease in cell proliferation (Figure 3C).

### PS-acet.-STAT3 peptide effectively suppresses human HCT116 tumor growth.

Our data showed an increased serum stability of PS-acet.-STAT3 peptide compared with its PO-modified counterpart in vitro and in vivo (Supplemental Figure 7). Further, in contrast to normal colorectal tissues, STAT3 acetylation was highly elevated in colorectal tumor tissues, as shown by fluorescent IHC staining of human colorectal tumor tissue array and 1 colorectal cancer (CRC) patient-derived xenograft (PDX) tumor tissue (Supplemental Figure 8, A and B, respectively). Next, the cell permeability of PS-acet.-STAT3 was examined and visualized in CRC patient primary tumor spheres. As shown in Supplemental Figure 8, C–E, in contrast to PO-acet.-STAT3 peptide, PS-acet.-STAT3 exhibited high cell permeability and significant inhibitory effects on cell proliferation and gene expression of STAT3 target genes in patient primary tumor spheres.

**Figure 4:**
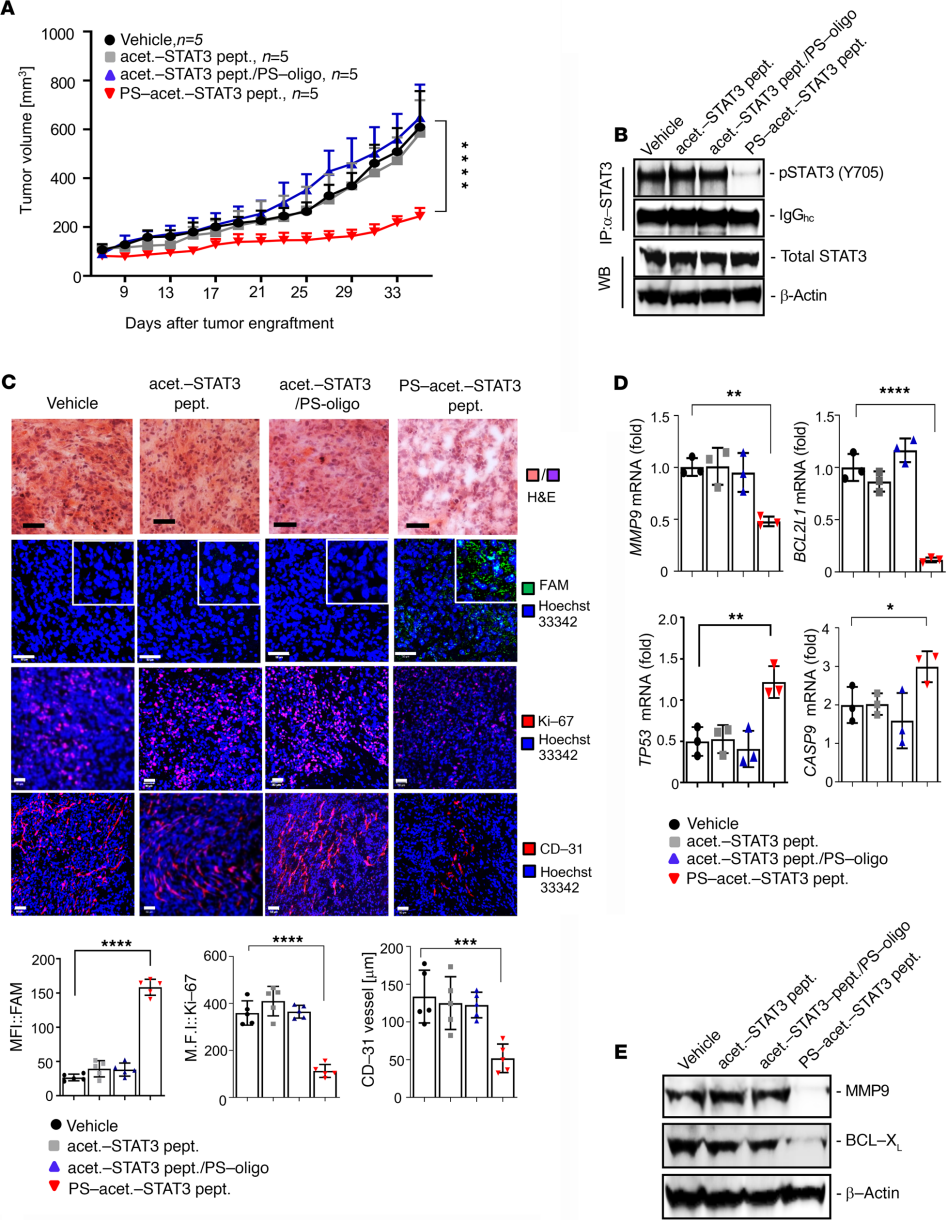
Treatments with PS-acet.-STAT3 peptide suppress growth of HCT116 xenograft tumors. (**A**) Growth kinetics of HCT116 tumors in NSG mice systemically treated with acet.-STAT3 peptides (1 mg/kg), with or without PS oligonucleotide conjugation, every other day (*n* = 5). SD is shown. Two-way ANOVA (Tukey’s multiple comparisons test) was used for analyzing the kinetics of tumor growth over the treatment period; ****P < 0.001. (**B**) Downregulation of tumor STAT3 activity in mice treated with PS-acet.-STAT3 peptide, as shown by immunoprecipitation followed by Western blotting. Lower 2 panels show input total STAT3 protein. (**C**) Treatment with PS-acet.-STAT3 peptide induces cell death and inhibits proliferation and angiogenesis in tumors, as indicated by confocal imaging and H&E staining (upper panel) and IF staining of Ki-67 and CD31 proteins in the tumor sections (lower panels). Penetration of FAM-labeled PS-acet.-STAT3 peptide into tumors was also assessed by immunostaining followed by confocal imaging (second panel from top). The images are representative of 5 tumors per experimental group in **A**. Scale bars: 50 μm. Insets: original magnification, ×20. The graphs show the quantification for FAM and Ki-67 expression levels and of the mean vessel diameter (data include 5 fields of view per group). SD is shown. One-way ANOVA; ****P < 0.001; ***P < 0.005. (**D**) Effects of downregulation of STAT3 activity on expression of proliferation- and apoptosis-related genes, as assessed by quantitative real-time RT-PCR in tumor homogenates from tumors shown in **A**. One-way ANOVA; ****P < 0.001; **P < 0.01; *P < 0.05. (**E**) Downregulation of STAT3 target proteins after treatments with PS-acet.-STAT3 peptide, analyzed by Western blotting using tumor homogenates from the tumors shown in **A**.

Therefore, we next tested the antitumor effects of PS-acet.-STAT3 peptide in HCT116 xenograft tumors. Immunodeficient *NOD/scid-IL-2Rγ_c_^null^* (NSG) mice were subcutaneously implanted with human HCT116 CRC cells and intravenously (i.v.) treated every other day with 1 mg/kg of PS-acet.-STAT3 peptide after tumors were established. Unconjugated acet-STAT3 peptide or unconjugated acet-STAT3-peptide plus PS-oligo (without chemical conjugation) was used as control. Our results showed that only PS-acet.-STAT3 peptide, but not the unconjugated control peptide or the vehicle, significantly reduced tumor growth after treatment (Figure 4A). Additionally, peritumoral treatments of PS-acet.-STAT3 peptides in HCT116 xenografted mice confirmed the antitumor effects of PS-acet.-STAT3 peptide, but not its PO-conjugated counterpart (Supplemental Figure 9A). Immunoprecipitation and Western blotting of tumor tissues showed that treatments with PS-acet.-STAT3 peptide downregulated STAT3 activation in HCT116 tumor xenografts (Figure 4B and Supplemental Figure 9, B and C).

Further, IF and confocal analyses revealed significant reduction of cell proliferation marker Ki-67 as well as tumor angiogenesis marker CD31 in tumor tissues upon treatments with PS-acet.-STAT3 peptide. Supporting the potent antitumor effects of PS-acet.-STAT3 peptide, we found massive cell death in tumor sections, as shown by the morphological features from H&E staining (Figure 4C). We also confirmed that STAT3 inhibition by PS-acet.-STAT3 peptide significantly reduced the expression of well-known STAT3 target genes in tumors (6), such as MMP-9 (*MMP9*) and BCL-X_L_ (*BCL2L1*) (Figure 4, D and E; and Supplemental Figure 9C). Furthermore, proapoptotic genes (*TP53*, *CASP9*) inhibited by STAT3 (5) were significantly upregulated in the tumor tissues treated with PS-acet.-STAT3 peptide (Figure 4D). Analyzing the homogenates from tumor tissues shown in Supplemental Figure 9A that were treated with PS-acet.-STAT3 peptide confirmed the decreased STAT3 activation and increased expression of apoptotic proteins, p53 and cleaved caspase-3, as well as the proapoptotic gene (*CASP9*), as analyzed by Western blotting and quantitative real-time PCR, respectively (Supplemental Figure 9, B, C, and E). Decreased cell proliferation and angiogenesis were shown in PS-acet.-STAT3 peptide–treated tumor tissues (Supplemental Figure 9D). Additionally, the overexpression of p53 in tumor tissues treated with PS-acet.-STAT3 peptide was confirmed with IF staining of tumor sections followed by confocal microscopy (Supplemental Figure 9F). To further confirm our in vitro data shown in Figure 1 that PS-acet.-STAT3 peptide could bind to exportin 7 to inhibit STAT3-exportin 7 interaction and further downregulate STAT3 activity, we performed immunoprecipitation of the FAM-labeled PS-acet.-STAT3 peptide or endogenous STAT3, followed by Western blotting with homogenates prepared from HCT116 colon tumors from mice treated with PO- and PS-acet.-STAT3 peptides. Consistent with our in vitro data, PS-acet.-STAT3 peptide bound to exportin 7 in tumor tissues and concomitantly blocked STAT3 binding to exportin 7 (Supplemental Figure 9G).

To evaluate the potential toxicity associated with systemic treatments of PS-peptide, PS-acet.-STAT3 peptide, PS-oligo, naive peptide, and PO- or PS-acet.-STAT3 peptide were given to the mice at 1 mg/kg through i.v. every other day for 3 weeks. No behavior changes, body weight loss, or abnormal blood counts were observed (Supplemental Figure 10). In addition, there was no significant morphology change or cell death in liver, spleen, or kidney tissues (Supplemental Figure 10).

### Dimerization of PS-acet.-STAT3 peptide enhances its antitumor efficacy.

**Figure 5:**
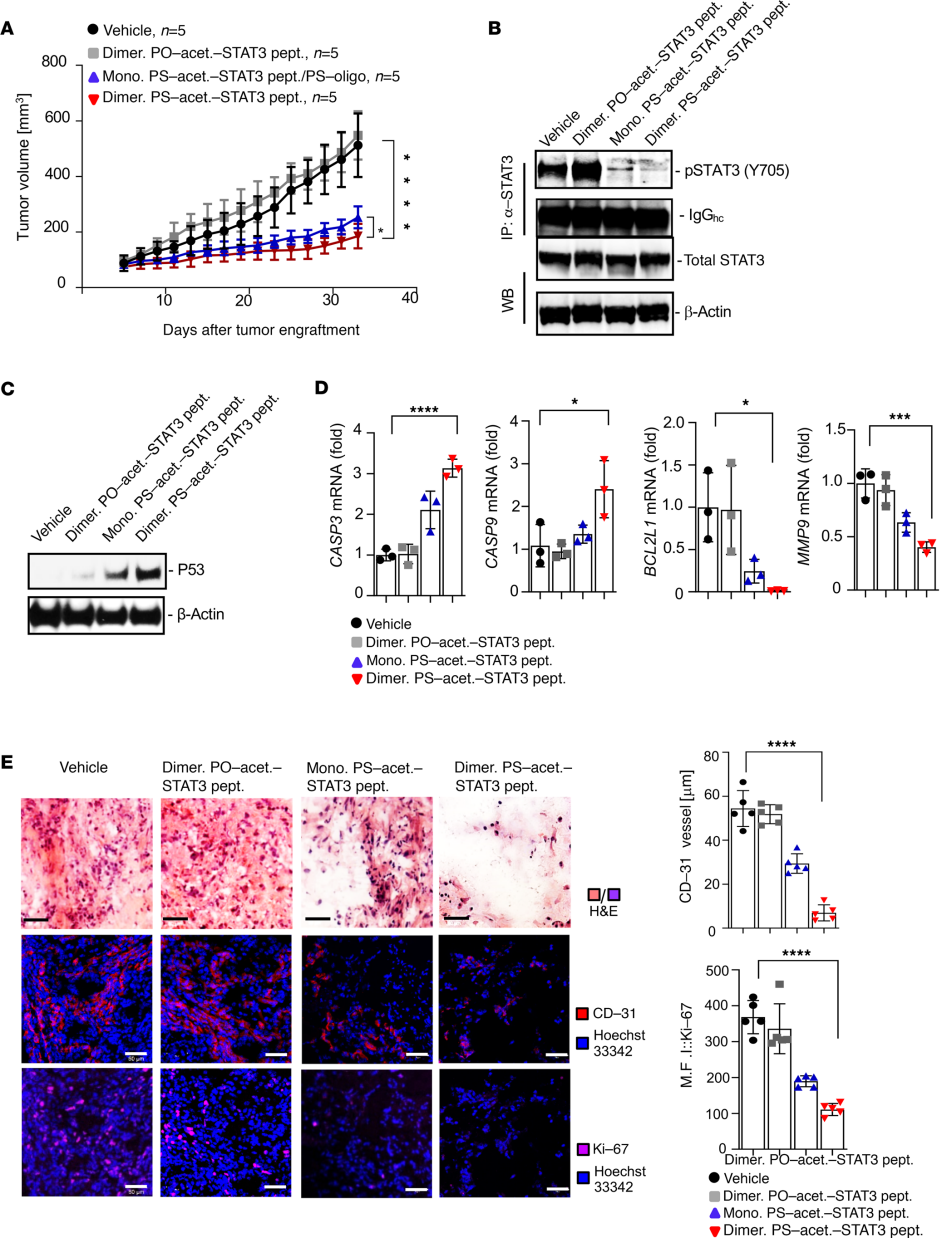
Dimerized PS-acet.-STAT3 peptide has improved antitumor functions. (**A**) The enhanced antitumor effect of the dimerized PS-acet.-STAT3 peptide is shown in HCT116 tumors grown in NSG mice (*n* = 5). Two-way ANOVA (Tukey’s multiple comparisons test) was used for analyzing the kinetics of tumor growth over the treatment period; ****P < 0.001; *P < 0.05. (**B**) The dimerized PS-acet.-STAT3 peptide downregulates pY705-STAT3 more effectively, as analyzed by immunoprecipitation and Western blotting with tumor homogenates from the tumors in **A**. hc, heavy chain of antibody. (**C**) The dimerized PS-acet.-STAT3 peptide induces p53 expression more effectively than its monomer counterpart, shown with Western blotting. (**D**) quantitative real-time RT-PCR showing mRNA levels of STAT3-regulated proapoptotic and prosurvival genes in HCT116 tumor tissues treated with the indicated STAT3 peptides in **A**. *n* = 3 independent quantitative real-time RT-PCR experiments. One-way ANOVA; ****P < 0.001; ***P < 0.005; *P < 0.05. (**E**) H&E and IF analysis of tumor sections from tumors shown in **A**. The antitumor effects of dimerized PS-acet.-STAT3 peptide were compared with its monomer counterpart. Images are representative of 5 tumors (*n* = 3) per experimental group. Scale bars: 50 μm (left panel). Right panels show the quantified Ki-67 protein levels as well as mean of vessel diameter (data include 5 fields of view per group). One-way ANOVA; ****P < 0.001.

Annealing 2 single-stranded oligonucleotides with complementary sequences conjugated on 2 separate peptides enabled dimerization of the modified PO- or PS peptides (Supplemental Figure 11). The efficiency of dimerization for both fluorescent-labeled PO and PS peptides was determined by nonreducing SDS-PAGE (Supplemental Figure 11B). We further assessed whether dimerized PS-acet.-STAT3 peptide can suppress tumor growth more effectively than its monomer counterpart in vivo. Immunodeficient NSG mice with established subcutaneous HCT116 xenograft tumors were treated with the monomer or dimerized PS-acet.-STAT3 peptides through peritumoral injection, as well as dimerized PO-acet.-STAT3 peptide or HBSS. Treatments with either monomer or dimerized PS-acet.-STAT3 peptides significantly reduced tumor growth. At the same time, there was further enhancement in antitumor efficacy with the dimerized PS-acet.-STAT3 peptides compared with its monomer counterpart (Figure 5A). Analyzing the tumor homogenates confirmed that tumor growth inhibition was due to a drastic decrease in STAT3 activity (Figure 5B) and increased tumor cell apoptosis (p53 overexpression) (Figure 5C). Furthermore, real-time quantitative PCR analysis indicated that dimerized PS-acet.-STAT3 peptide, relative to its monomer counterpart, more effectively suppressed expression of STAT3 positively regulated genes (*MMP9* and *BCL2L1*) while upregulating expression of the proapoptotic genes, *CASP3* and *CASP9* (Figure 5D). Notably, confocal microscopy following IF staining confirmed a considerable decrease in proliferation and angiogenesis markers (Ki-67 and CD31) and massive cell death (H&E staining) in tumors treated with the dimerized PS-acet.-STAT3 peptide, compared with not only PO-acet.-STAT3 peptide but also its monomer counterpart (Figure 5E).

### PS-acet.-STAT3 peptide induces antitumor immunity.

**Figure 6:**
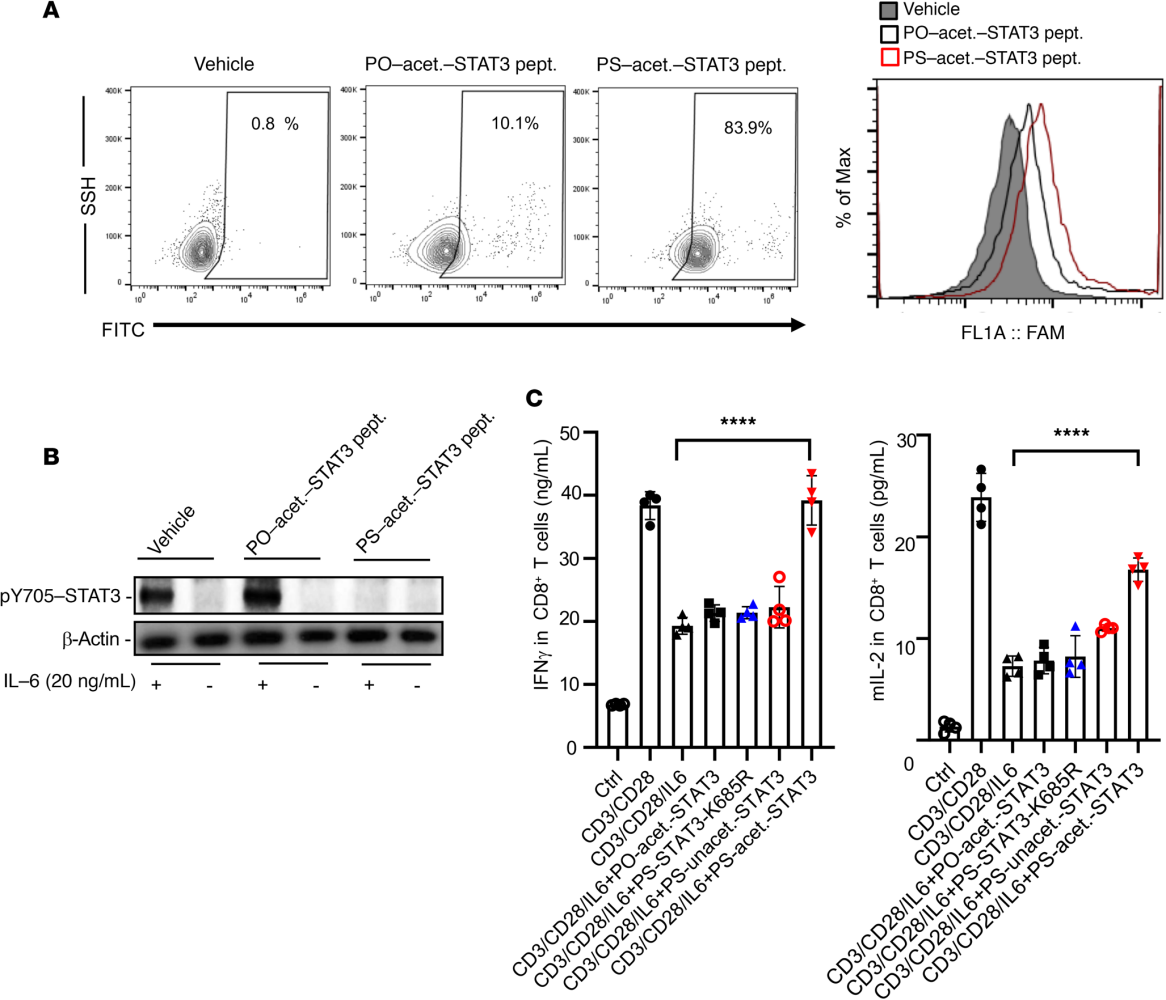
PS-acet.-STAT3 peptide treatment increases CD8^+^ T cell cytotoxicity. (**A**) In vitro penetration of PS-acet.-STAT3 peptide into CD8^+^ T cells, as assessed by flow cytometry. CD8^+^ T cells were isolated from C57BL/6 mouse spleens. Data are representative of 3 independent experiments (*n* = 3). (**B**) Downregulation of IL-6–induced pY705-STAT3 in splenic CD8^+^ T cells by PS-acet.-STAT3 peptide compared with controls, as examined by Western blotting. (**C**) ELISA shows that treating activated splenic CD8^+^ T cells with PS-acet.-STAT3 peptide upregulates IFN-γ and IL-2 expression, as expected from STAT3 inhibition. Data represent 4 independent experiments (*n* = 4). SD is shown. One-way ANOVA; ****P < 0.001. Assays were done in triplicates. mIL-2, murine IL-2.

It has been extensively documented that STAT3 is also persistently activated in tumor-associated immune cells (6, 8). STAT3 in both myeloid cells and T cells promotes expression of immunosuppressive factors while inhibiting many Th1 immunostimulatory molecules (8). To test whether PS-acet.-STAT3 peptide could penetrate and inhibit STAT3 activity in T cells, we incubated PO- and PS-acet.-STAT3 peptides with freshly isolated mouse splenic CD8^+^ T cells in vitro. Flow cytometric analysis confirmed the efficient internalization of PS-acet.-STAT3 peptide in splenic CD8^+^ T cells, compared with its PO-conjugated counterpart (Figure 6A). To mimic tumor microenvironment, IL-6, an immunosuppressive cytokine and potent STAT3 activator, was added to freshly isolated splenic CD8^+^ T cells to activate STAT3 and consequently to inhibit their cytotoxicity. As we expected, IL-6–induced STAT3 activation (pY705 phosphorylation) was downregulated by PS-acet.-STAT3 peptide treatment in the CD8^+^ T cells (Figure 6B). Next, we tested the ability of PS-acet.-STAT3 peptide in regulating immunostimulatory cytokine production in CD8^+^ T cells by ELISA. Mouse splenetic T cells were isolated and activated by anti-CD3 and anti-CD28 antibodies. IL-6 was added to mimic tumor microenvironment. To increase scientific rigor and highlight the important role of acetylation of STAT3 in immune modulation, PS-unacet.-STAT3 and PS-STAT3-K685R peptides were included as control peptides. The results indicate that only PS-acet.-STAT3 peptide treatment induced immunostimulatory cytokines interferon-γ (IFN-γ) and IL-2 production by CD8^+^ T cells (Figure 6C). Our results confirmed that PS-acet.-STAT3 peptide penetrated into the CD8^+^ T cells, inhibited STAT3 activity, and further promoted their antitumor immunity.

**Figure 7:**
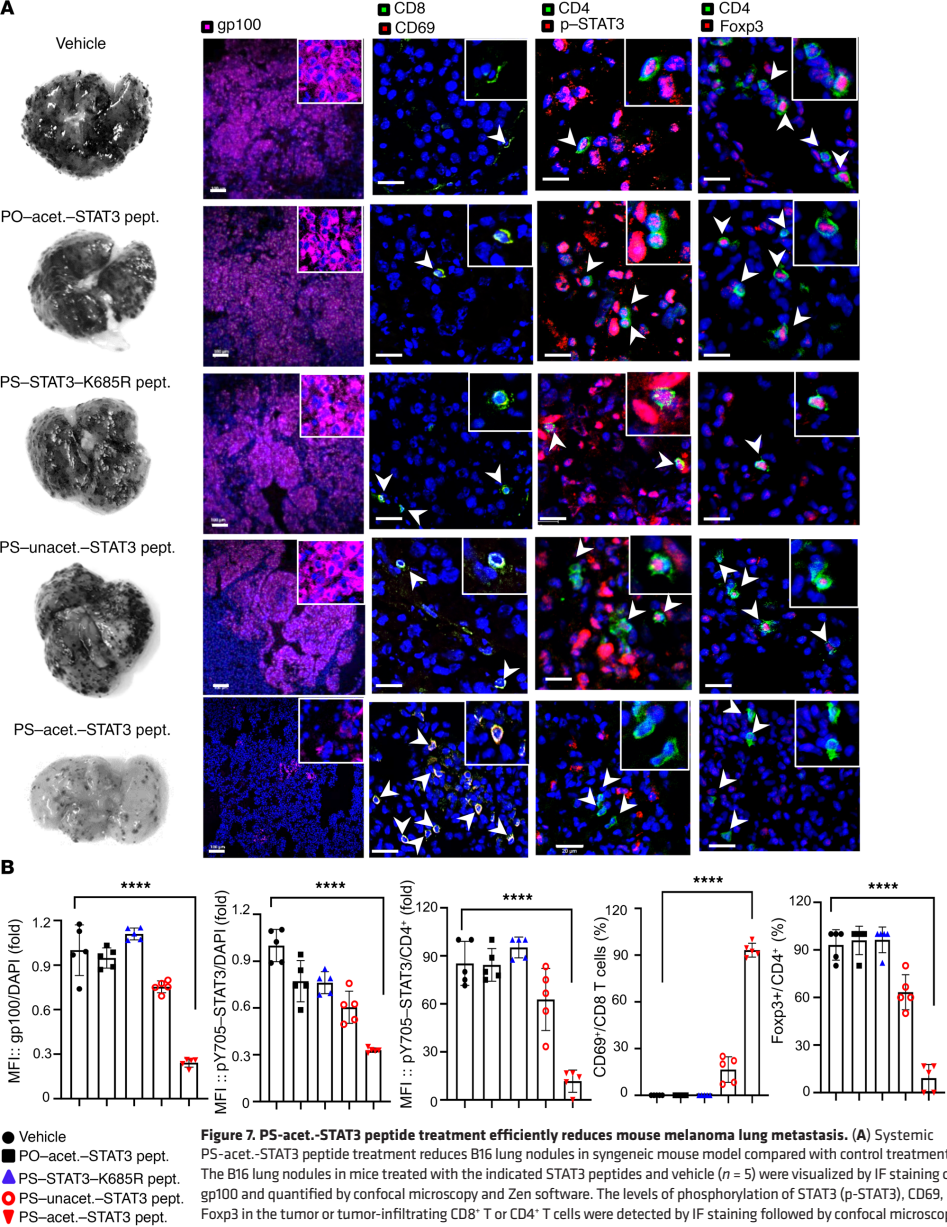
PS-acet.-STAT3 peptide treatment efficiently reduces mouse melanoma lung metastasis. (**A**) Systemic PS-acet.-STAT3 peptide treatment reduces B16 lung nodules in syngeneic mouse model compared with control treatments. The B16 lung nodules in mice treated with the indicated STAT3 peptides and vehicle (*n* = 5) were visualized by IF staining of gp100 and quantified by confocal microscopy and Zen software. The levels of phosphorylation of STAT3 (p-STAT3), CD69, and Foxp3 in the tumor or tumor-infiltrating CD8^+^ T or CD4^+^ T cells were detected by IF staining followed by confocal microscopy in B16 metastatic lung nodules upon treatments with vehicle, PO- or PS-acet.-STAT3, PS-STAT3-K685R, or PS-unacet.-STAT3 peptides as indicated. Images are representative of 5 lungs per experimental group. Scale bars: 20 μm (CD69, CD8, CD4, p-STAT3, and Foxp3) and 100 μm (gp100). White arrows indicate the immune cells. Insets: original magnification, ×40. (**B**) The graphs show the quantification of gp100 and p-STAT3 expression levels (MFI) or the cell population (%) of CD69^+^ in CD8^+^ or p-STAT3^+^ in CD4^+^ or Foxp3^+^ in CD4^+^ (data represent 5 lungs per group, *n* = 5). SD is shown. One-way ANOVA; ****P < 0.001.

We next tested to what extent PS-acet.-STAT3 peptide could affect antitumor immune responses in a syngeneic mouse tumor model. Immune-competent C57BL/6 mice were intravenously challenged with the mouse B16 melanoma cells, followed by i.v. injection of PS-unacet.-STAT3, PS-STAT3-K685R, and PO- or PS-acet.-STAT3 peptides, as well as vehicle. Mouse B16 melanoma is a murine tumor cell line used for the study of lung metastasis and immune surveillance (48). Systemic treatments with PS-acet.-STAT3 peptide led to a striking reduction of metastatic lung nodules. On the contrary, few antitumor effects in groups treated with PS-unacet.-STAT3, PS-STAT3-K685R, PO-Ac-STAT3, or vehicle were observed (Figure 7A). The tumor tissue sections were analyzed by IF staining and confocal microscopy imaging, which showed that treatments with PS-acet.-STAT3 peptide significantly decreased STAT3 activation (p-STAT3) in tumor cells and CD4^+^ T cells and increased the infiltration of CD69^+^ activated CD8^+^ T cells in metastatic B16 lung nodules (Figure 7B) (49). Conversely, tumor-associated FoxP3^+^CD4^+^ T cells (regulatory T cells) that induce immunosuppression and promote tumor progression and metastasis (50, 51) were concomitantly reduced (Figure 7B).

### Blocking STAT3 axis by PS-Gp130 and MYC peptides inhibits tumor growth and increases tumor infiltration of cytotoxic CD8^+^ T cells in mouse pancreatic cancer model.

Pancreatic ductal adenocarcinoma (PDAC) is one of the deadliest malignant diseases with very poor clinical outcome, and patients with PDAC do not respond well to current immunotherapies. To further illustrate the broad use of PS cell-penetrating peptide approach to tackle the most challenging cancer and targets, we further tested a Gp130 and a MYC peptide. Elevated IL-6 plays an important role in PDAC metastasis and progression. IL-6 binds to α-receptor IL-6R to form a binary complex (IL-6/IL-6Rα), which is further dimerized with Gp130. The IL-6/IL-6Rα/Gp130 complex activates JAKs, which in turn activates STAT3 (5, 6, 52). Persistent STAT3 activation has been reported in 30% to 100% of human PDAC tumor specimens (53–55). In addition to IL-6/Gp130/STAT3 signaling pathway, elevated activities of transcription factor MYC are associated with poor prognosis of patients with PDAC (56, 57). However, to date, there have been no direct MYC inhibitors. MYC heterodimerizes with MAX, and this dimer binds to E-box sequences in the promoters of numerous oncogenic genes (58, 59). In addition to its role in oncogenesis, MYC has been shown to regulate the tumor microenvironment through effects on both innate and effector cells and immune-regulatory cytokines (60, 61).

**Figure 8:**
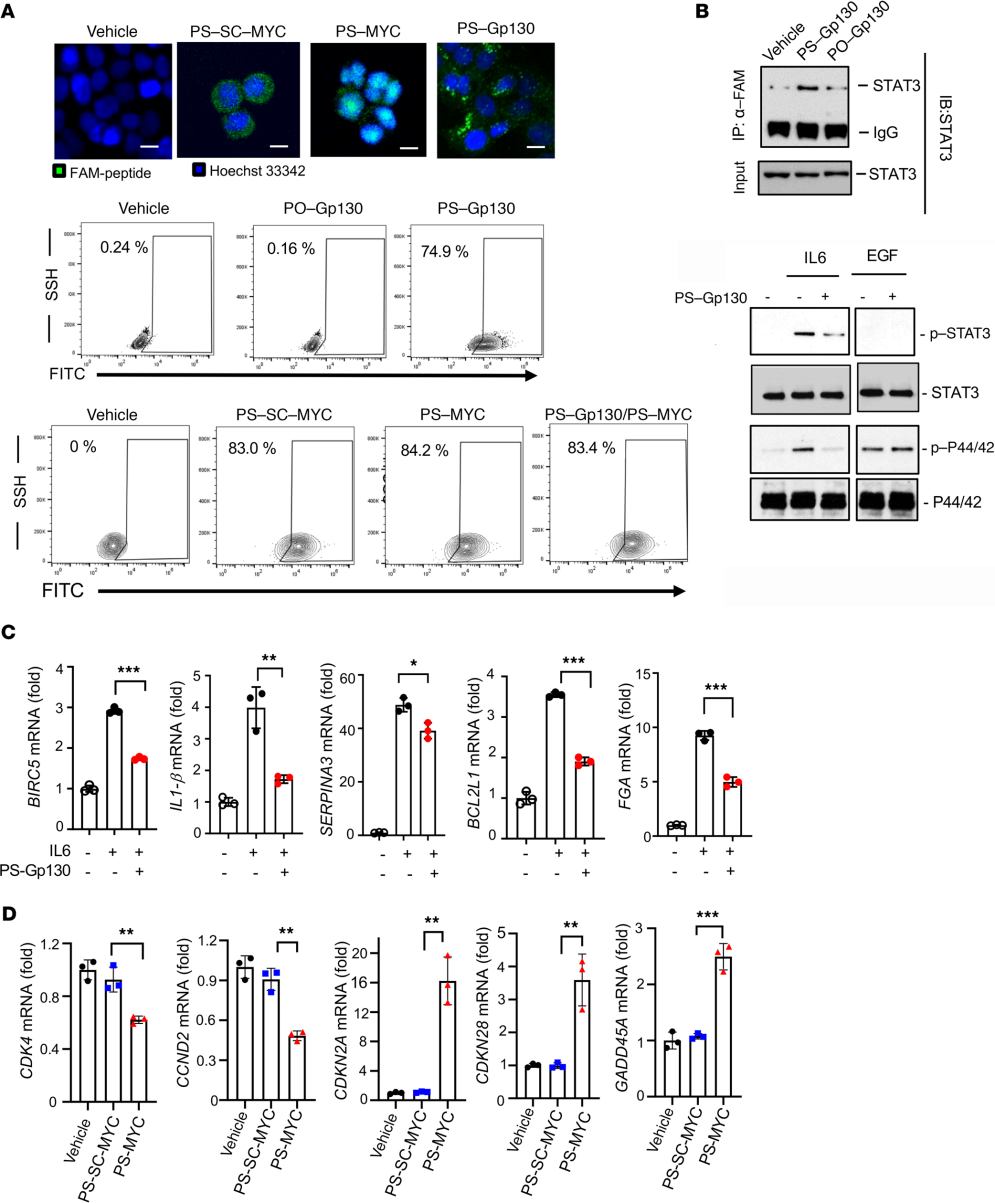
PS-Gp130 and PS-MYC blocking peptides efficiently internalize into tumor cells to exert their functions. (**A**) For 1 hour 1 μM of PS-conjugated MYC (PS-MYC) and Gp130 (PS-Gp130) were incubated with cancer cells. PS-scramble MYC (PS-SC-MYC) was used as a control for PS-MYC. The peptide uptake was visualized by confocal microscopy and quantified by flow cytometry. Data are representative of 3 independent experiments (*n* = 3). (**B**) Upper panel: The binding of PS-Gp130 and STAT3 was examined by immunoprecipitation and Western blotting. PO-Gp130 was used as a control. For 4 hours prior to harvest, 1 μM of peptide was incubated with cells. Lower panel: PS-Gp130 inhibits IL-6–induced phosphorylation of STAT3 and p44/42 but not EGF-mediated signaling. The cells were preincubated with PS-Gp130 for 2 hours and then stimulated with 20 ng/mL of IL-6 or 30 ng/mL of EGF for 30 minutes before harvest and Western blotting. (**C**) Gene expression of STAT3 targets was examined by quantitative PCR after the treatment with 1 μM of PS-Gp130 with or without IL-6 stimulation as described above. Data shown are 3 independent experiments (*n* = 3); SD is shown. Unpaired Student’s *t* test; ***P < 0.005; **P < 0.01; *P < 0.05. (**D**) A total of 1 μM of PS-SC-MYC or PS-MYC was incubated with cells for 48 hours before the gene expression of MYC targets was examined by quantitative PCR. Data shown are 3 independent experiments (*n* = 3). SD is shown. Unpaired Student’s *t* test; ***P < 0.005; **P < 0.01; *P < 0.05.

We assessed whether inhibiting IL-6/Gp130 and MYC through cell-penetrating decoy peptides (Supplemental Figure 12A) could block the protein-protein interactions between MYC and MAX, and between Gp130-STAT3, respectively. PS-MYC peptide corresponds to a segment of leucine zipper of MAX (amino acids 77–85), which is necessary for MYC and MAX heterodimerization and DNA binding (59). A PS-conjugated peptide with scrambled amino acid sequence of MYC peptide (PS-SC-MYC) was designed to be used as a control. PS-Gp130 spans 10 amino acids, representing the motif of STAT3 binding and activation (amino acids 901–910) (1, 62). Because phosphorylation of Tyr905 in Gp130 has been shown to be essential for STAT3 activation, a phospho-group was added to Tyr905 in PS-Gp130 peptide to facilitate STAT3 binding. As shown in Figure 8A, PS conjugation enabled highly efficient cell penetration of both PS-MYC and PS-Gp130 peptides in cancer cells. Confocal microscopy also revealed PS conjugation facilitated not only peptide penetration but also target recognition. PS-MYC peptide but not PS-SC-MYC peptides were retained in the nucleus, where MYC-MAX heterodimer resides. PS-Gp130 was detected in the cytoplasm or associated with the membrane (Figure 8A). The results from immunoprecipitation and Western blotting indicated that PS-Gp130 peptide was strongly bound to STAT3 (Figure 8B, upper panel) and selectively inhibited IL-6–induced STAT3 phosphorylation but not EGF-mediated ERK phosphorylation (Figure 8B, lower panel). STAT3 signaling was also significantly blocked by PS-Gp130 peptide treatment (Figure 8C). PS-MYC peptide treatment also markedly downregulated the expression of MYC-mediated cell cycle and survival genes while upregulating the expression of MYC-suppressed target genes (Figure 8D).

**Figure 9:**
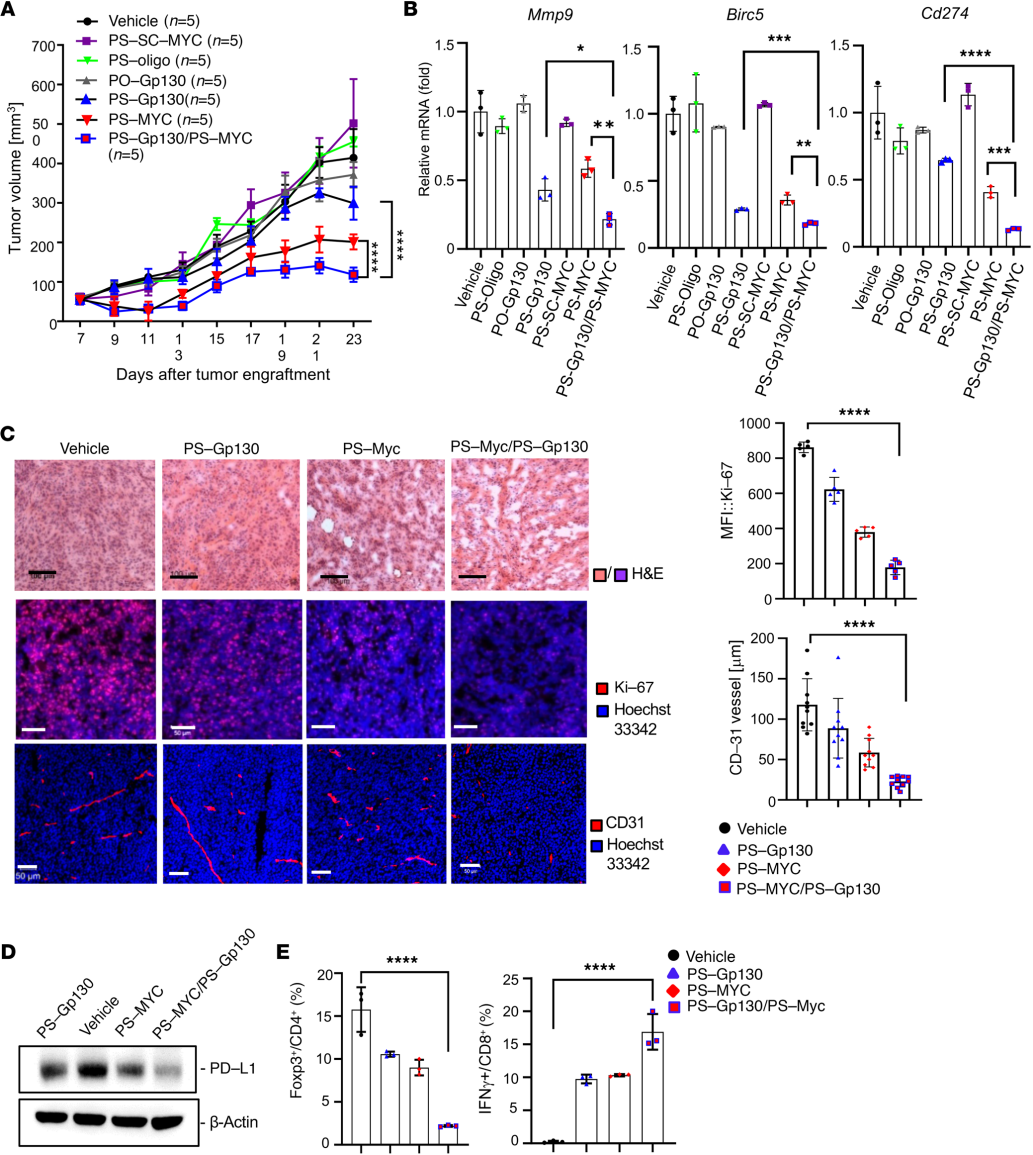
Blocking Gp130 and c-MYC significantly increases tumor infiltration of cytotoxic CD8^+^ T cells in mouse pancreatic cancer model. (**A**) Pancreatic KPC tumor cells were subcutaneously xenografted in C57BL/6 mice. The tumors were systemically treated with PS-peptides as indicated (*n* = 5 per group) every other day at 1 mg/kg. Combination of PS-MYC and PS-Gp130 peptides was given at 0.5 mg/kg, respectively. SD is shown. Two-way ANOVA. ****P < 0.0001. (**B**) Quantitative PCR showing the levels of *Mmp9*, *Cd274*, and *Birc5* in pooled tumor tissues treated with the indicated peptides in **A**. *n* = 3 independent quantitative PCR experiments. SD is shown. Unpaired Student’s *t* test; ****P < 0.001; ***P < 0.005; **P < 0.01; *P < 0.05. (**C**) H&E and IF analysis of tissue sections from the tumors treated as indicated in **A**. Images are representative of 5 tumors per experimental group. H&E, scale bars: 100 μm. IF, scale bars: 50 μm. Right panels show the quantified Ki-67 level and the mean of vessel diameter (CD31; data include 2 fields of view per lung section). SEM is shown. One-way ANOVA; ****P < 0.001. (**D**) The expression of PD-L1 in the pooled tumor tissues that were treated as indicated was examined by Western blotting. (**E**) The population of IFN-γ^+^ in CD8^+^ T or Foxp3^+^ in CD4^+^ T cells in the pooled tumor tissues treated as indicated was quantified by flow cytometry. Data shown are mean of 3 replicates (*n* = 3). SD is shown; 1-way ANOVA; ****P < 0.001.

We next tested to what extent PS-MYC and PS-Gp130 peptides could affect antitumor immune responses in a syngeneic mouse PDAC tumor model. KPC mouse PDAC cells were subcutaneously inoculated in immune-competent C57BL/6 mice, followed by i.v. injection of PS-MYC, PS-SC-MYC, and PO- or PS-Gp130 peptides, as well as PS-oligo or vehicle. We found PS-MYC or PS-Gp130 peptide treatments significantly inhibited PDAC tumor growth. Importantly, combined PS-MYC and PS-Gp130 peptide treatment markedly further promoted mouse PDAC tumor regression (Figure 9A). Compared with individual PS-MYC or PS-Gp130 peptide treatment, the expression of IL-6/Gp130/STAT3 axis and MYC target genes was modulated to a greater extent with the combined treatment (Figure 9B). Further, IF and confocal analyses revealed further reduction of cell proliferation marker Ki-67 and tumor angiogenesis marker CD31 in tumor tissues upon combined treatment with PS-MYC and PS-Gp130 peptides (Figure 9C). Supporting the potent antitumor effects of combined peptide treatment, we found massive cell death in tumor sections, as shown by the morphological features from H&E staining (Figure 9C).

In addition to antitumor effects through suppressing cell proliferation and angiogenesis, we found that combined treatment with PS-MYC and PS-Gp130 peptides remarkably decreased the expression level of programmed cell death ligand 1 (PD-L1) in mouse PDAC tumors (Figure 9D) while activating tumor-infiltrating CD8^+^ T cells, as shown by IFN-γ production (Figure 9E). Furthermore, combined treatment with PS-MYC and PS-Gp130 peptides also suppressed regulatory CD4^+^ T cell activation (Foxp3^+^) (Figure 9E). These results suggest that blocking IL-6/Gp130/STAT3-MYC axis by PS-Gp130 and PS-MYC peptides can induce PDAC tumor regression through both antiproliferation/survival of tumor cells and induction of antitumor immune responses.

## Discussion

The current study offers a potentially novel strategy to improve peptide-based therapies through increasing cell penetrability. Our recently published work demonstrated that attaching PS DNA oligonucleotides to antibodies enables their cell penetration into cultured cells without interfering with the antibody’s antigen recognition, leading to target blockade and regulation of the target downstream genes (36). PS DNA oligonucleotide–modified antibodies also efficiently penetrated into cells within tumors through both local and systemic administrations. By conjugating a PS DNA oligonucleotide to a synthetic acetylated STAT3 (K685), MYC, or Gp130 peptide, we show PS modification can also facilitate efficient penetration of peptide into tumor cells and tumor tissues in vivo. Like the cell-penetrating antibodies using PS DNA oligonucleotide modification (36), internalization of PS peptides is affected by membrane potential polarity. This is likely only a partial mechanism underlying PS DNA oligonucleotide modification–mediated peptide cellular internalization. As we pointed out in the publication on the mechanism of PS antibody cell internalization, using PS DNA oligonucleotides to modify proteins to enable their cellular entry stemmed from an unexpected observation, not by design. Due to the lack of general knowledge about the permeability barrier properties of cell membranes, it is a challenge to design experiments to fully identify the mechanisms underlying protein cellular internalization by PS DNA oligonucleotide attachment.

One unique feature of the cell-penetrating antibody is that binding of the antibody to its intracellular target facilitates its cellular retention. Without a target to bind, PS antibodies egress and are not detectable in cultured cells and tumors. Similarly, we found that PS-acet.-STAT3 peptide, but not PS-STAT3-KR or PS-unacet.-STAT3 peptide, was effectively accumulated in tumor cells and in tumors in which acetyl-STAT3 level was elevated. Our cell-penetrating peptides were biologically effective at markedly lower concentration relative to other peptides, including improved stapled cyclic (25, 26) or D-peptides (27). For in vivo treatments in animal tumor models, PS-acet.-STAT3, PS-MYC, or PS-Gp130 peptide was given at 1 mg/kg whereas the stapled p53 peptides were administered at 10 mg/kg (23). Another intracellular therapeutic peptide, Omomyc, is also under the spotlight. Omomyc is a dominant-negative variant of MYC with mutations that can form heterodimers with WT MYC and consequently block the binding of MYC to MAX. However, its in vivo antitumor effective dosage is at 2.37 mg/kg (compared with 1 mg/kg of PS-acet.-STAT3, PS-MYC or PS-Gp130 peptide) (63, 64). Although serum stability is low for a native peptide, several methods have shown to improve peptide stability (65, 66). As an added benefit, we found the serum stability of PS-conjugated peptide in vivo was prolonged compared with its PO-modified counterpart. While PS-modified oligonucleotides themselves are more stable than their unmodified counterparts (67, 68), why adding 1 PS DNA oligonucleotide to each peptide is sufficient to protect the peptide from serum degradation remains to be explored. Consequently, the in vivo antitumor effects could be contributed by both increased penetrability and serum stability. As we show in the current study, PS DNA oligonucleotide modification enables creation of bispecific/function peptides, by virtue of the complementary nature of attached nucleic acids on the individual peptide.

Our studies further provide proof of concept that a potentially novel STAT3 inhibitor specifically blocks STAT3 at K685, leading to potent antitumor effects in multiple tumor models. Increasing evidence suggests that acetylation of STAT3 at K685 contributes to its oncogenic activity (33, 34) and that elevated acetylation of STAT3 at K685 has been detected in a variety of human cancers (3). We and others have also reported that genetically altering STAT3 at K685 inhibits tumor progression (34), suggesting that acetylated STAT3 is a valid target to develop anticancer therapeutics. Our results here validated K685 at STAT3 is a crucial target to inhibit tumor cell growth/survival and elicit antitumor immune responses. In the current study, we only examined penetrability of PS-peptide into T cells as shown with isolated murine CD8^+^ T cells. However, STAT3 is known to be activated in many types of immune cells in the tumor microenvironment (6, 8, 69). Downregulation of CD4^+^ T regulatory cells and upregulation of CD8^+^ T cells within tumors in syngeneic, immune-competent mice could be contributed by inhibition of STAT3 by PS-acet.-peptide in myeloid cells, B cells, and DCs.

One of the major concerns is that MYC and STAT3 are involved in many essential functions of the biology of normal cells, so potent MYC and STAT3 inhibitors might be associated with pronounced toxicities. However, current in vivo data argue that systemic MYC or STAT3 inhibition is possible. For example, only mild toxicities in tissues were observed upon systemic MYC inhibition by Omomyc in mice (64). In addition, a STAT3 inhibitor, napabucasin, already passed phase I and II clinical trials and currently is in a phase III clinical trial (42, 70, 71). Those animal experiments and clinical trials argue for an existing therapeutic window for MYC and STAT3 (42, 64). In our systemic toxicity test in vivo, PS-peptide treatments did not cause mouse body weight loss, behavior changes, or abnormal blood counts (Supplemental Figures 10 and 12). Although minor anemia is suggested from the blood count in the group of mice treated with PS-MYC peptide, it was one of the expected on-target effects as MYC is required for hematopoiesis. However, anemia is a common side effect for many clinically used anticancer chemotherapies. Understandably, third party–performed comprehensive toxicity studies are required before clinical translation. Our preliminary laboratory toxicity studies support further evaluation of PS-peptide for possible clinical translation.

Taken together, we have extended our cell-penetrating antibody platform to create peptides with high cellular penetrability. Using this platform, we developed potentially novel PS-conjugated STAT3, MYC, and Gp130 peptides that effectively and specifically block activated STAT3 axis and MYC function in tumor cells and in tumor microenvironment. These peptides may have the potential to be developed into efficacious clinical anticancer therapeutics.

## Methods

### Cell culture.

Murine B16 melanoma, human HCT116 colon carcinoma, human LoVo metastatic CRC, and U251 human glioma cell lines were purchased from American Type Culture Collection and cultured according to vendor’s instructions. KPC mouse cell line was a gift from Haiyong Han (Molecular Medicine Division, TGen, Phoenix, Arizona, USA) and maintained in DMEM supplied with 10% FBS.

### Oligopeptide structure and synthesis.

The modified peptides were produced by Anaspec and Eurogenetec.

### Peptide hybridization.

PO- and PS-acet.-STAT3 peptides with a complementary sense or antisense DNA strand were mixed 1:1, boiled for 2 minutes at 65°C, and cooled down slowly to room temperature (around 3–4 hours). Peptides were then stored at –20°C. Dimerization was confirmed by running samples on a nonreducing 4%–12% gradient Bis-Tris gel (Invitrogen, Thermo Fisher Scientific) using NuPAGE MES SDS running buffer (Invitrogen, Thermo Fisher Scientific). To visualize peptides, a ChemiDoc imager (Bio-Rad) was used.

### In vivo experiments.

Mouse care and experimental procedures were performed under pathogen-free conditions in accordance with established institutional guidance and approved IACUC protocols from Research Animal Care Committee of the City of Hope. For s.c. tumor challenge, 5 × 10^6^ HCT116 cells were injected into NSG mice from The Jackson Laboratory or animal resource core facility at the City of Hope. After tumors reached an average size of 80~100 mm^3^, mice were locally or intravenously treated with 1 mg/kg of PO- or PS-peptides (Anaspec) every day or every other day. Control animals were treated with the equal volume of HBSS (Invitrogen, Thermo Fisher Scientific). Tumor growth was measured by a digital caliper every other day. To study the effects of the modified peptides at protein and gene levels, mice were euthanized 4–6 weeks after tumor injection. B16 melanoma lung metastasis was performed by injecting 0.5 × 10^6^ B16 cells into bloodstream of C57BL/6 mice (The Jackson Laboratory). One day after tumor challenge, mice were treated intravenously (retro-orbitally) with 1 mg/kg of PO- or PS-acet.-STAT3, PS-unacet.-STAT3, or PS-STAT3-K685R peptides every other day until the endpoint as indicated. Control mice were treated with HBSS. Lungs were excised from the mice, and B16 nodules were visualized by gp100 staining. Lungs were then analyzed for immune responses by immunostaining followed by confocal imaging. For mouse PDAC model, 5 × 10^6^ KPC mouse cells were mixed with Matrigel and subcutaneously injected into C57BL/6 mice (The Jackson Laboratory). After tumors reached an average size of 80~100 mm^3^, mice were intravenously treated with 1 mg/kg of PO- or PS-Gp130, PS-oligo, PS-SC-MYC, PS-MYC. or combined PS-Gp130/PS-MYC (0.5 mg/kg, 0.5 mg/kg) (Anaspec) every other day for 18 days. Control animals were treated with the equal volume of HBSS (Invitrogen, Thermo Fisher Scientific). Tumor growth was measured by a digital caliper every other day. To study the effects of the modified peptides at protein and gene levels, mice were euthanized at the endpoint as indicated.

### Immunoblotting and immunoprecipitation.

Whole cell lysates or tissue homogenates were prepared by using RIPA lysis buffer (50 mM Tris at pH 7.4, 150 mM NaCl, 1 mM EDTA, 0.5% NP-40, 1 mM NaF, 15% glycerol, and 20 mM β-glycerophosphate). A protease inhibitor cocktail (Mini Protease Inhibitor Cocktail; Roche) and a tyrosine phosphatase inhibitor, sodium orthovanadate (Na_3_VO_4_, 1 mM), were added freshly to the lysis buffer. Protein amounts were determined by BCA using a Thermo Fisher Scientific kit. Normalized protein amounts were subjected to an SDS-PAGE and transferred onto a nitrocellulose membrane for Western blotting. Proteins were visualized with a chemiluminescent detection kit (Pierce, Thermo Fisher Scientific). The protein levels for STAT1 (sc-346, E-23), STAT3 (sc-482, C-20), STAT5 (sc-74442, A-9), Crm1 (exportin 1) (sc-5595, H-300), CAS (exportin 2) (sc-271537, H-2), exportin 5 (sc-66885, H-300), and exportin 7 (sc-98639, H-82) were determined using antibodies from Santa Cruz Biotechnology Inc. Anti–exportin 3 (exportin T, 630622, LOS1 05-1525), anti–exportin 4 (3499-1), and anti–exportin 6 (11408-1-AP) were from MilliporeSigma, Epitomics, and Proteintech, respectively. In addition, primary antibodies against STAT3 (catalog 9139, 124H6), p-STAT3 (Tyr705) (catalog 9131), BCL-X_L_ (catalog 2764, 54H6), p53 (catalog 4667, 7F5), cleaved caspase-3 (catalog 9654), p44/42 MAPK (catalog 9102), p-p44/42 MAPK (catalog 9101), c-MYC (catalog 5605, D84C12), MMP9 (catalog 13667, D6O3H), and Survivin (catalog 2808, 71G4B7) were purchased from Cell Signaling Technology. Anti-FITC (also binds to FAM) (catalog 71-1900) was from Invitrogen, Thermo Fisher Scientific, and anti-actin (clone AC-74; A2228) from MilliporeSigma. HRP-streptavidin secondary detection reagent was from MilliporeSigma (RAB-HRP3). For immunoprecipitation, protein G agarose beads (Invitrogen, Thermo Fisher Scientific) were coated with 2 μg/sample anti-FITC antibody (also binds to FAM) (catalog 71-1900) (Invitrogen, Thermo Fisher Scientific) or anti-STAT3 antibody (sc-482, C-20) (Santa Cruz Biotechnology) and incubated with cell lysates or tumor homogenates at 4°C overnight. Samples were subjected to SDS-PAGE followed by Western blotting.

### Luciferase reporter gene assay.

A total of 1–2 × 10^6^ HCT116 cells were seeded into each well of a 24-well plate (Cellstar) and transfected with a 13x RGC plasmid, with a Renilla luciferase construct (both plasmids provided by Bernhard Lüscher, RWTH, Aachen, Germany), using Lipofectamine 2000 (Invitrogen, Thermo Fisher Scientific), according to manufacturer’s instruction. On the following day, cells were treated with 1 μM of PO- and PS-acet.-STAT3 peptides at 37*°*C overnight. Luciferase activities were determined using the luciferase assay system (Promega).

### IF staining.

IF staining was carried out by seeding 0.5 × 10^5^ U251 cells on coverslips in a 24-well plate (Cellstar) and incubating with 1 μM of PO- or PS-acet.-STAT3 peptides for 30 minutes at 37°C. Cells were fixed for 20 minutes with 2% paraformaldehyde (Electron Microscopy Sciences), permeabilized for 5 minutes with PBS containing 0.1% Triton X-100 (PBS-T), quenched with 50 mM NH_4_Cl in PBS-T, and blocked with 1% BSA (MilliporeSigma) in PBS-T. Immunostaining was performed using an anti-STAT3 antibody (sc-482, C-20, Santa Cruz Biotechnology). The nuclei were also stained with Hoechst 33342 (MilliporeSigma). For immunohistofluorescence (IHC), the tumor tissues were collected, and part of the tissues were embedded in OCT block. The tissue sections from the OCT-frozen tumor tissues were fixed in 2% formaldehyde, permeabilized with methanol, and blocked in PBS containing 10% goat, rat, or rabbit serum (MilliporeSigma). Samples were stained at 4°C overnight with primary antibodies against CD31 (BD Pharmingen, clone MEC, 550274), p53 (Santa Cruz Biotechnology, clone DO-1, sc-126), Ki-67 (Cell Signaling Technology, D3B5, 12202), FITC (also binds to FAM) (Abcam, ab19224), Foxp3 (Abcam, ab54501), and CD69 (Abcam, H1.2F3, ab25190). Anti-CD8 (clone 53-6.7, 100703) and anti-CD4 (clone GK1.5, 100403) were purchased from BioLegend. Anti-mouse PD-L1 (rabbit; D5V3B; 64988) antibody was purchased from Cell Signaling Technology. IFN-γ antibody (JM10-10) was from Novus Biologicals (NBP2-66900).

The fluorophore secondary antibodies were from Invitrogen, Thermo Fisher Scientific, unless noted: donkey anti-goat, Alexa Fluor 488 (A-11055); donkey anti-rabbit, Alexa Fluor 647 (A32795TR); donkey anti-rat, DyLight 550 (SA5-10027); donkey anti-rabbit, DyLight 550 (SA5-10039); goat anti-rabbit IgG antibody (H+L), biotinylated (BA-1000-1.5, Vector Laboratories); goat anti-hamster IgG antibody (H+L), biotinylated (BA-9100-1.5, Vector Laboratories); Cy2 streptavidin (016-220-084, Jackson ImmunoResearch Laboratories, Inc.); and Cy3 streptavidin (016-160-084, Jackson ImmunoResearch Laboratories, Inc.). The nuclei were stained with Hoechst 33342 (MilliporeSigma). After staining with antibodies, slides were mounted and analyzed by confocal microscopy. The confocal imaging was carried out using a ×40 or ×20 immersion objective on LSM 700 confocal microscope (ZEISS). Images were analyzed and quantified using Zen software (ZEISS).

### Cell proliferation assay.

HCT116 cells were seeded in a 96-well plate at a density of 6000/well. Starting the following day, cells were treated every day with 10 μg/mL (approximately 1 μM) PO- or PS-peptides. On day 7, cell proliferation was measured based on ATP production using bioluminescence detection kit for ATP measurement (CellTiter-Glo, Promega), according to manufacturer’s instruction.

### Quantitative real-time PCR.

Total RNA was extracted from cultured or tumor tissues using RNeasy kit (QIAGEN), according to manufacturer’s instruction. After cDNA synthesis using iScript cDNA Synthesis kit (Bio-Rad), samples were analyzed using pairs of primers specific for *CASP9*, *CASP3*, *CASP8*, *BIRC5*, *BCL2L1*, *MYC*, *TP53*, *VEGF*, *IL1B*, *SERPIN3*, *FGA*, *CDK4*, *CCND2*, *CDKN2A*, *CDKN2B*, *GADD45A*, *mCD274*, *mMmp9*, and *mBirc5*, as indicated. Sequence-specific amplification was detected by fluorescence signal of SYBR Green (Bio-Rad) by using Chromo4 real-time PCR detector (Bio-Rad). Real-time PCR was performed in triplicate using the Chromo4 real-time detector (Bio-Rad). The human *GAPDH* or *ACTB* or mouse *Actb* housekeeping gene was used as an internal control to normalize target gene mRNA levels. Primers are from Integrated DNA Technologies: human caspase-9: *hCASP9*: F: 5′-TGTCCTACTCTACTTTCCCCAGGT TTT-3′, R: 5′-GTGAGCCCACTGCTCAAAGAT-3′; human caspase-8: *hCASP8*: F: 5′-ATGCAAACTGGATGATGACA-3′ R: 5′-GATTATCTTCAGCAGGCTCTT-3′; human caspase-3: *hCASP3*: F: 5′-ACATGGCGTGTCATAAAATACC-3′, R: 5′-CACAAAGCGACTGGATGAAC-3′; human p53: *hTP53*: F: 5′-GCGAGCACTGCCCAACAACA-3′, R: 3′-GGATCTGAAGGGTGAAATATTCT-5′; human BCL-X_L_: *hBcl2l1*: F: 5′-GTCCTCACTCCCAGTCCAA-3′, R: 5′-GCTGAGGCCATAAACAGC TC-3′; human Survivin: *hBIRC5*: F: 5′-TCCCTGGCTCCTCTACTGTT-3′, R: 5′-TGTCTCCTCATCCACCTGAA-3′; human MYC: *MYC*: F: 5′-CACCGAGTCGTAGTCGAGGT-3′, R: 5′-GCTGCTTAGACGCTGGATTT-3′; human MMP9: *MMP9*: F: 5′-TTGACAGCGACAAGAAGTGG-3′, R: 5′-GCCATTCACGTCGTCCTTAT-3′; human β-actin: *hACTB*: F: 5′-AGGCACCAGGGCGTGAT-3′, R: 5′-GCCCACATAGGAATCCTTCTGAC-3′; human *IL1B*: F: 5′-TTCGACACATGGGATAACGA-3′, R: TCTTTCAACACGCAGGACAG-3′; human *SERPIN3*; F: 5′-CTCAGTCTGCTGGACAGGTT-3′, R: 5′-TGAGTATCTTGGGGGTCAAA-3′; human *FGA*, F: 5′-GAGAGGCCATGCTAAATCTC-3′, R: AACTTAGTCTAGGGGGACAG-3′; human *CDK4*, F: 5′-GTCGGCTTCAGAGTTTCCAC-3′, R: 5′-CCGAAGTTCTTCTGCAGTCC-3′; human *CCND2*: F: 5′-TCATTGAGCACATCCTTCGCAAGC-3′, R: 5′-GGCAAACTTGAAGTCGGTAGCACA-3′; human *CDKN2A*, F: 5′-CAACGCACCGAATAGTTACGG-3′, R: 5′-AACTTCGTCCTCCCAGAGGTCGC; human *CDKN2B*, F: 5′-GTGAGAGTGGCAGGGTCTG-3′, R: 5′-GTACAGGAGTCTCCGTTGGC-3′; human *GADD45A*, F: 5′-GCAGGATCCTTCCATTGAGA-3′, R: 5′-CTCTTGGAGACCGACGCTG-3′; mouse *Cd274*, F: 5′-CCACGGAAATTCTCTGGTTG-3′, R: 5′-TGCTGCATAATCAGCTACGG-3′; mouse *Mmp9*, F: 5′-GCTGACTACGATAAGGACGGCA-3′, R: 5′-TAGTGGTGCAGGCAGAGTAGGA-3′; mouse *Birc5*, F: 5′-GAACCCGATGACAACCCGAT-3′, R: 5′-TGGCTCTCTGTCTGTCCAGT-3′; mouse *Actb*, F: 5′-CATTGCTGACAGGATGCAGAAGG-3′, R: 5′-TGCTGGAAGGTGGACAGTGAGG-3′.

### Isolation of CD8^+^ T cells.

Spleens from C57BL/6 mice were excised, gently minced and incubated in 400 U/mL of Collagenase D/DNAse I solution (Roche) for 15 minutes at 37°C. Cell suspensions were filtered through a mesh filter (Falcon, Corning). RBCs were lysed, using an RBC lysis buffer (MilliporeSigma) and the cells were resuspended in 10 mL of HBSS containing 2% FBS. CD8^+^ T cells were isolated using MojoSort mouse CD8^+^ T cell isolation kit (BioLegend), according to manufacturer’s instruction.

### Peptide penetration.

Peptide penetration assays were performed on tumor cell lines (1–2 × 10^5^/mL) or CD8^+^ T cell suspension (0.7 × 10^6^/mL) from C57BL/6 spleens. Cells were treated with 1 μM of FAM-labeled PO- or PS-conjugated peptides for 15 minutes to 2 hours at 37*°*C. To study the mechanism of internalization, cells were pretreated with 120 mM KCl to induce depolarization of the cell membrane, followed by adding 1 μM FAM-labeled PO- or PS-acet.-STAT3 peptides. Bis-(1,3-Dibutylbarbituric Acid) Trimethine Oxonol [DiBAC_4_(3)] (Thermo Fisher Scientific) was applied as a positive control according to manufacturer’s instructions. Fluorescence data were collected on Accuri C6 (BD Biosciences) or Fortessa (BD Biosciences) and analyzed using FlowJo software (Tree Star).

### Retention experiments in vitro and in vivo.

K685R STAT3 mutant (34) or STAT3 WT HCT116 cells were treated with 10 μg/mL (approximately 1 μM) FAM-labeled PO- or PS-acet.-STAT3 peptide. After 4 hours’ incubation at 37°C, medium was replenished. The intensity of FAM-labeled peptide in the cells was assessed 16 hours after replenishment of culture medium with Attune NxT Cytometer (Thermo Fisher Scientific).

For in vivo study, HCT116 cells were subcutaneously engrafted into NSG mice. After tumors reached an average size of approximately 5 mm, mice were locally treated with 1 mg/kg of FAM-labeled PS-unacet.-STAT3, PS-STAT3-K685R, or PS-acet.-STAT3 peptides (Anaspec) every day for 35 days. Twenty-four hours after the last treatment, tumors were harvested and analyzed by IF staining and 20× immersion objective on LSM 700 confocal microscope (ZEISS). Images were quantified using Zen software (ZEISS).

### Peptide biostability in vitro and in vivo.

First, 25% (*v/v*) of human or mouse serum (MilliporeSigma) in HBSS was temperature-equilibrated at 37°C for 15 minutes. Then, 5 μg of FAM-labeled PO- or PS-acet.-STAT3 peptide was added to temperature-equilibrated serum. Samples were collected after 0, 30, 60, and 120 minutes as well as overnight and run on 15% Bis-Tris gels using NuPAGE MES SDS Running Buffer (Invitrogen, Thermo Fisher Scientific). To visualize FAM-labeled peptides, a ChemiDoc imager (Bio-Rad) was used.

To study the biostability of PS-acet.-STAT3 peptide in vivo, HCT116 tumor-bearing NSG mice were systemically treated with 1 mg/kg FAM-labeled acet.-STAT3 peptide (with or without PS conjugation) every other day. Twenty-four hours after the last treatment, the whole blood was collected. Blood was allowed to clot by leaving at room temperature for 15–30 minutes. Samples were centrifuged at 1000*g* for 10 minutes. The supernatants (serum) were collected and analyzed on a 10% Bis-Tris gel using NuPAGE MES buffer (Invitrogen, Thermo Fisher Scientific). The FAM-labeled peptide was detected with a ChemiDoc imager (Bio-Rad). Albumin was visualized by Coomassie staining (Invitrogen, Thermo Fisher Scientific) and detecting the gel with a ChemiDoc imager (Bio-Rad).

### CD8^+^ T cell activation and ELISA.

CD8^+^ T cells were isolated from C57BL/6 mice spleens as described above. A 96-well plate (Cellstar) was precoated with 2 μg/mL anti-CD3 antibody (BioLegend, clone 145-2c11, 100331) at 4°C overnight (BioLegend), and 1–2 × 10^5^ cells were seeded into each well, followed by adding 2 μg/mL soluble CD28 (BioLegend, clone 37-51, 102012) and stimulation with 20 ng/mL IL-6. PS-unacet.-STAT3, PS-STAT3-K685R, and PO- or PS-acet.-STAT3 peptides at a concentration of 1 μM were added to the cells. Peptide treatment was repeated on the following day. Control cells were treated with HBSS. After 48 hours’ treatment with peptides at 37°C, supernatants were assessed for IFN-γ and IL-2 production by R&D, Bio-Techne, kits (DY402 and DY406), according to manufacturer’s instruction.

### Flow cytometry analysis of immune cells from PDAC tumor tissues.

Five PDAC tumor tissues harvested from each treatment group were pooled (half of the tumor tissue) and excised, gently minced, and incubated in 400 U/mL of Collagenase D/DNAse I solution (Roche) for 15 minutes at 37°C. Cell suspensions were filtered through a mesh filter (Falcon, Corning). After RBCs were lysed by RBC lysis buffer (MilliporeSigma), the cells were resuspended in 1 mL of HBSS containing 2% FBS (staining buffer) and 1 μM monensin (GolgiStop, BD Biosciences) to retain intracellular cytokine. The cells were subsequently stained for surface markers (anti-CD8 and anti-CD4 antibodies as described above) on ice for 30 minutes. For intracellular staining, cells were subsequently washed in staining buffer twice before fixation and permeabilization using eBioscience’s buffers (Foxp3 staining kit, Thermo Fisher Scientific). After that, the cells were stained for IFN-γ and Foxp3 and analyzed on Attune NxT flow cytometry instrument (Thermo Fisher Scientific).

### Statistics.

All in vitro experiments were done at least 3 times. Statistical analyses were performed using Prism (GraphPad) software. Two-tailed unpaired Student’s *t* test was used to calculate statistical differences between any 2 groups. One-way ANOVA was used to calculate statistical differences between any 3 or more groups. Two-way ANOVA (Tukey’s multiple comparisons test) was used for analyzing the kinetics of tumor growth and STAT3 protein stability. *P* values of less than 0.05 were considered statistically significant.

### Study approval.

Animal care and experimental approaches with mice were performed under pathogen-free conditions in accordance with established institutional guidance and approved IACUC protocols (10003 and 08026) from the Research Animal Care Committee of the City of Hope.

## Author contributions

MA designed and conducted most PS-acet.-STAT3 peptide studies and interpreted the data; YJL designed and performed B16, CRC PDX, and PDAC studies and data interpretation; QZ contributed to in vitro and in vivo experiments; CZ provided general guidance with all the immunological experiments; JS, NA, TN, and CL contributed to in vitro studies. MF provided PDX tissues and general guidance for the CRC study; DKA provided general guidance for the CRC study; AH and HY were the major contributors to the development of the cell-penetrating platform and provided overall guidance for the study; MA, YJL, and HY prepared and wrote the manuscript along with the help from all the coauthors.

## Supplementary Material

Supplemental data

## Version 1 01/25/2021

Electronic publication

## References

[1] Hirano T (2000). Roles of STAT3 in mediating the cell growth, differentiation and survival signals relayed through the IL-6 family of cytokine receptors. Oncogene.

[2] Bromberg J (2000). The role of STATs in transcriptional control and their impact on cellular function. Oncogene.

[3] Yu H, Jove R (2004). The STATs of cancer--new molecular targets come of age. Nat Rev Cancer.

[4] Johnson DE (2018). Targeting the IL-6/JAK/STAT3 signalling axis in cancer. Nat Rev Clin Oncol.

[5] Yu H (2014). Revisiting STAT3 signalling in cancer: new and unexpected biological functions. Nat Rev Cancer.

[6] Yu H (2009). STATs in cancer inflammation and immunity: a leading role for STAT3. Nat Rev Cancer.

[7] Kujawski M (2008). Stat3 mediates myeloid cell-dependent tumor angiogenesis in mice. J Clin Invest.

[8] Yu H (2007). Crosstalk between cancer and immune cells: role of STAT3 in the tumour microenvironment. Nat Rev Immunol.

[9] Zhang J (2009). Targeting cancer with small molecule kinase inhibitors. Nat Rev Cancer.

[10] Zhang X (2013). A novel inhibitor of STAT3 homodimerization selectively suppresses STAT3 activity and malignant transformation. Cancer Res.

[11] Furtek SL (2016). Strategies and approaches of targeting STAT3 for cancer treatment. ACS Chem Biol.

[12] Kettner NM (2019). Combined inhibition of STAT3 and DNA repair in palbociclib-resistant ER-positive breast cancer. Clin Cancer Res.

[13] Debnath B (2012). Small molecule inhibitors of signal transducer and activator of transcription 3 (Stat3) protein. J Med Chem.

[14] Yang CL (2012). Curcumin blocks small cell lung cancer cells migration, invasion, angiogenesis, cell cycle and neoplasia through Janus kinase-STAT3 signalling pathway. PLoS One.

[15] Tu SP (2012). Curcumin induces the differentiation of myeloid-derived suppressor cells and inhibits their interaction with cancer cells and related tumor growth. Cancer Prev Res (Phila).

[16] Fossey SL (2011). The novel curcumin analog FLLL32 decreases STAT3 DNA binding activity and expression, and induces apoptosis in osteosarcoma cell lines. BMC Cancer.

[17] Yang YP (2012). Resveratrol suppresses tumorigenicity and enhances radiosensitivity in primary glioblastoma tumor initiating cells by inhibiting the STAT3 axis. J Cell Physiol.

[18] Lin CL (2012). Protective effect of caffeic acid on paclitaxel induced anti-proliferation and apoptosis of lung cancer cells involves NF-κB pathway. Int J Mol Sci.

[19] Hong D (2015). AZD9150, a next-generation antisense oligonucleotide inhibitor of STAT3 with early evidence of clinical activity in lymphoma and lung cancer. Sci Transl Med.

[20] Leong PL (2003). Targeted inhibition of Stat3 with a decoy oligonucleotide abrogates head and neck cancer cell growth. Proc Natl Acad Sci U S A.

[21] Kortylewski M (2009). In vivo delivery of siRNA to immune cells by conjugation to a TLR9 agonist enhances antitumor immune responses. Nat Biotechnol.

[22] Moreira D (2018). STAT3 inhibition combined with CpG immunostimulation activates antitumor immunity to eradicate genetically distinct castration-resistant prostate cancers. Clin Cancer Res.

[23] Chang YS (2013). Stapled α-helical peptide drug development: a potent dual inhibitor of MDM2 and MDMX for p53-dependent cancer therapy. Proc Natl Acad Sci U S A.

[24] Wang E (2019). Tumor penetrating peptides inhibiting MYC as a potent targeted therapeutic strategy for triple-negative breast cancers. Oncogene.

[25] Baek S (2012). Structure of the stapled p53 peptide bound to Mdm2. J Am Chem Soc.

[26] Bernal F (2007). Reactivation of the p53 tumor suppressor pathway by a stapled p53 peptide. J Am Chem Soc.

[27] Liu M (2010). D-peptide inhibitors of the p53-MDM2 interaction for targeted molecular therapy of malignant neoplasms. Proc Natl Acad Sci U S A.

[28] Meric-Bernstam F (2017). Phase I trial of a novel stapled peptide ALRN-6924 disrupting MDMX- and MDM2-mediated inhibition of WT p53 in patients with solid tumors and lymphomas. J Clin Oncol.

[29] Ren Z (2003). Identification of a high-affinity phosphopeptide inhibitor of Stat3. Bioorg Med Chem Lett.

[30] Yue P, Turkson J (2009). Targeting STAT3 in cancer: how successful are we?. Expert Opin Investig Drugs.

[31] Turkson J (2004). Novel peptidomimetic inhibitors of signal transducer and activator of transcription 3 dimerization and biological activity. Mol Cancer Ther.

[32] Auzenne EJ (2012). A phosphopeptide mimetic prodrug targeting the SH2 domain of Stat3 inhibits tumor growth and angiogenesis. J Exp Ther Oncol.

[33] Yuan ZL (2005). Stat3 dimerization regulated by reversible acetylation of a single lysine residue. Science.

[34] Lee H (2012). Acetylated STAT3 is crucial for methylation of tumor-suppressor gene promoters and inhibition by resveratrol results in demethylation. Proc Natl Acad Sci U S A.

[35] Yang J (2010). Reversible methylation of promoter-bound STAT3 by histone-modifying enzymes. Proc Natl Acad Sci U S A.

[36] Herrmann A (2019). An effective cell-penetrating antibody delivery platform. JCI Insight.

[37] Zhuang S (2013). Regulation of STAT signaling by acetylation. Cell Signal.

[38] Herrmann A (2014). STAT3 nuclear egress requires exportin 7 via engaging lysine acetylation. MOJ Cell Sci Rep.

[39] Grothey A (2017). CanStem303C trial: a phase III study of napabucasin (BBI-608) in combination with 5-fluorouracil (5-FU), leucovorin, irinotecan (FOLFIRI) in adult patients with previously treated metastatic colorectal cancer (mCRC). J Clin Oncol.

[40] Shah MA (2016). The BRIGHTER trial: a phase III randomized double-blind study of BBI-608+weekly paclitaxel versus placebo (PBC)) plus weekly paclitaxel in patients (pts) with pretreated advanced gastric and gastro-esophageal junction (GEJ) adenocarcinoma. J Clin Oncol.

[41] Sonbol MB (2019). CanStem111P trial: a phase III study of napabucasin plus nab-paclitaxel with gemcitabine. Future Oncol.

[42] Kawazoe A (2020). Phase 1 study of napabucasin, a cancer stemness inhibitor, in patients with advanced solid tumors. Cancer Chemother Pharmacol.

[43] Li Y (2015). Suppression of cancer relapse and metastasis by inhibiting cancer stemness. Proc Natl Acad Sci U S A.

[44] Carpenter RL, Lo HW (2014). STAT3 target genes relevant to human cancers. Cancers (Basel).

[45] Bromberg J (2002). Stat proteins and oncogenesis. J Clin Invest.

[46] Niu G (2005). Role of Stat3 in regulating p53 expression and function. Mol Cell Biol.

[47] Srivastava SK, Sahu RP (2009). Response: re: the role of STAT-3 in the induction of apoptosis in pancreatic cancer cells by benzyl isothiocyanate. J Natl Cancer Inst.

[48] Overwijk WW, Restifo NP (2001). B16 as a mouse model for human melanoma. Curr Protoc Immunol.

[49] Testi R (1989). T cell activation via Leu-23 (CD69). J Immunol.

[50] Takeuchi Y, Nishikawa H (2016). Roles of regulatory T cells in cancer immunity. Int Immunol.

[51] Saito T (2016). Two FOXP3(+)CD4(+) T cell subpopulations distinctly control the prognosis of colorectal cancers. Nat Med.

[52] Bollrath J (2009). gp130-mediated Stat3 activation in enterocytes regulates cell survival and cell-cycle progression during colitis-associated tumorigenesis. Cancer Cell.

[53] Corcoran RB (2011). STAT3 plays a critical role in KRAS-induced pancreatic tumorigenesis. Cancer Res.

[54] Panni RZ (2014). Tumor-induced STAT3 activation in monocytic myeloid-derived suppressor cells enhances stemness and mesenchymal properties in human pancreatic cancer. Cancer Immunol Immunother.

[55] Lesina M (2011). Stat3/Socs3 activation by IL-6 transsignaling promotes progression of pancreatic intraepithelial neoplasia and development of pancreatic cancer. Cancer Cell.

[56] Sancho P (2015). MYC/PGC-1α balance determines the metabolic phenotype and plasticity of pancreatic cancer stem cells. Cell Metab.

[57] Schleger C (2002). c-MYC activation in primary and metastatic ductal adenocarcinoma of the pancreas: incidence, mechanisms, and clinical significance. Mod Pathol.

[58] Amati B (1993). Oncogenic activity of the c-Myc protein requires dimerization with Max. Cell.

[59] Nair SK, Burley SK (2003). X-ray structures of Myc-Max and Mad-Max recognizing DNA. Molecular bases of regulation by proto-oncogenic transcription factors. Cell.

[60] Casey SC (2018). The MYC oncogene is a global regulator of the immune response. Blood.

[61] Casey SC (2016). MYC regulates the antitumor immune response through CD47 and PD-L1. Science.

[62] Novotny-Diermayr V (2002). Protein kinase C delta associates with the interleukin-6 receptor subunit glycoprotein (gp) 130 via Stat3 and enhances Stat3-gp130 interaction. J Biol Chem.

[63] Soucek L (2002). Omomyc, a potential Myc dominant negative, enhances Myc-induced apoptosis. Cancer Res.

[64] Beaulieu ME (2019). Intrinsic cell-penetrating activity propels Omomyc from proof of concept to viable anti-MYC therapy. Sci Transl Med.

[65] Gentilucci L (2010). Chemical modifications designed to improve peptide stability: incorporation of non-natural amino acids, pseudo-peptide bonds, and cyclization. Curr Pharm Des.

[66] Chan LY (2011). Engineering pro-angiogenic peptides using stable, disulfide-rich cyclic scaffolds. Blood.

[67] Iwamoto N (2017). Control of phosphorothioate stereochemistry substantially increases the efficacy of antisense oligonucleotides. Nat Biotechnol.

[68] Miller CM, Harris EN (2016). Antisense oligonucleotides: treatment strategies and cellular internalization. RNA Dis.

[69] Kortylewski M, Yu H (2007). Stat3 as a potential target for cancer immunotherapy. J Immunother.

[70] Jonker DJ (2014). A phase I extension study of BBI608, a first-in-class cancer stein cell (CSC) inhibitor, in patients with advanced solid tumors. J Clin Oncol.

[71] Bendell J (2017). Phase 1b/II study of cancer stemness inhibitor napabucasin in combination with FOLFIRI plus /-evacizumab (bev) in metastatic colorectal cancer (mCRC) patients (pts). Annals Oncology.

