# Supplementary material for: Potent antitumor effects of cell-penetrating peptides targeting STAT3 axis
Source: JCI Insight. 2021 Jan 25;6(2):e136176. doi: 10.1172/jci.insight.136176 (PMC7934871; doi:10.1172/jci.insight.136176)
Supplement: Supplemental data [file jciinsight-6-136176-s155.pdf]

PS-unacet.-STAT3 peptide

PDIPKEEAFGKYCRPESQEHPC-linker-T*C*C*A*T*G*A*G*C*T*T*C*C*T*G*A*T*G*C*T

PS-STAT3-K685R peptide

PDIPKEEAFGRYCRPESQEHPC-linker-T*C*C*A*T*G*A*G*C*T*T*C*C*T*G*A*T*G*C*T

PS-acet.-STAT3 peptide

PDIPKEEAFGK685(Ac)YCRPESQEHPC-linker-T*C*C*A*T*G*A*G*C*T*T*C*C*T*G*A*T*G*C*T

PO-acet.-STAT3 peptide

PDIPKEEAFGK685(Ac)YCRPESQEHPC-linker-TCCATGAGCTTCCTGATGCT

Supplemental Figure 1. Amino acid sequences of phosphorothioated (*, PS) and/or non-phosphorothioated (PO) DNA oligonucleotide-modified STAT3 peptides. PS modified STAT3 peptides span 22 amino acids of STAT3 protein (amino acids 675-697) including Lysine 685 (K685). By adding an acetyl group to lysine 685, acetyl-STAT3 peptide (PS-acet.-STAT3 pept.) was created. The mutant STAT3 peptide contains arginine instead of lysine 685 (PS-STAT3-K685R pept.). One peptide with the same amino acid sequence without acetylation and mutation was designed as PS-unacet.-STAT3 peptide. PO-acet.-STAT3 is acetyl-STAT3 peptide conjugated to non-phosphorothioated polymer backbone.

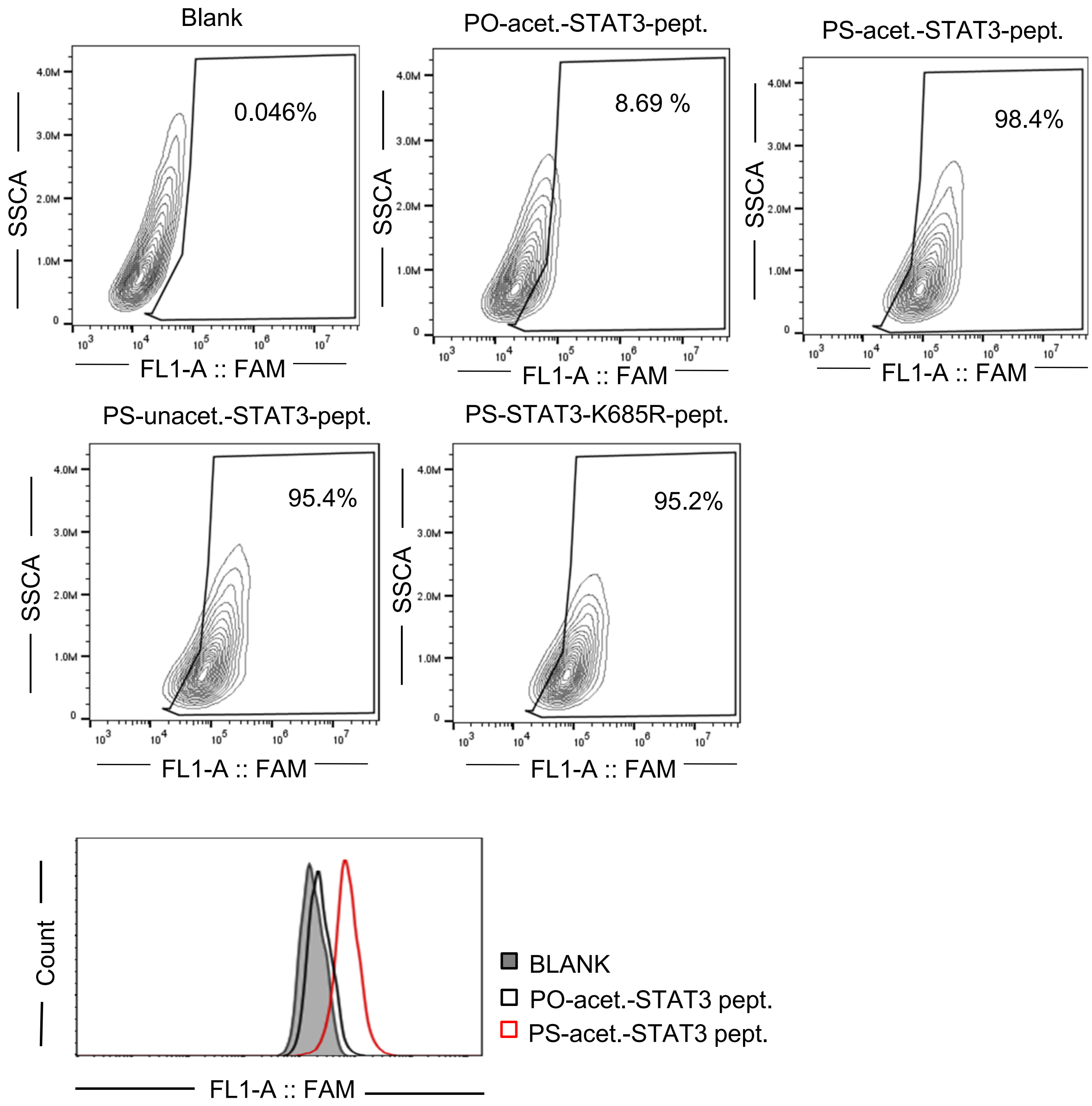

Supplemental Figure 2 (related to Figure 1). The cell penetration efficiency (%) and fluorescence intensity of phosphorothioated DNA oligonucleotide-modified STAT3 peptide (FAM-labeled) in human glioma cell line U251, as assessed by flow cytometry. Data shown is the representative of 3 independent experiments.

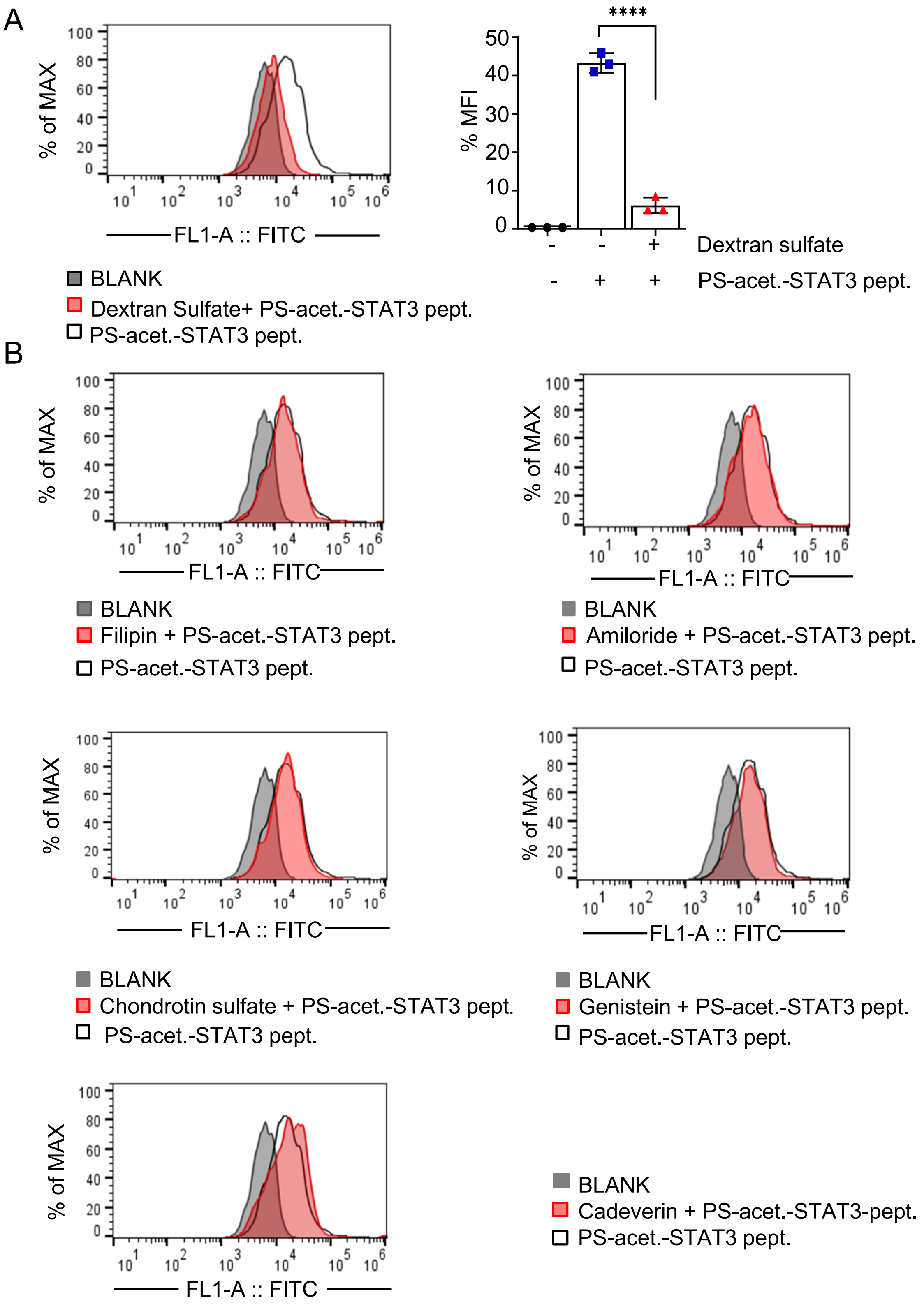

Aftabizadeh and Li et al., Figure S3

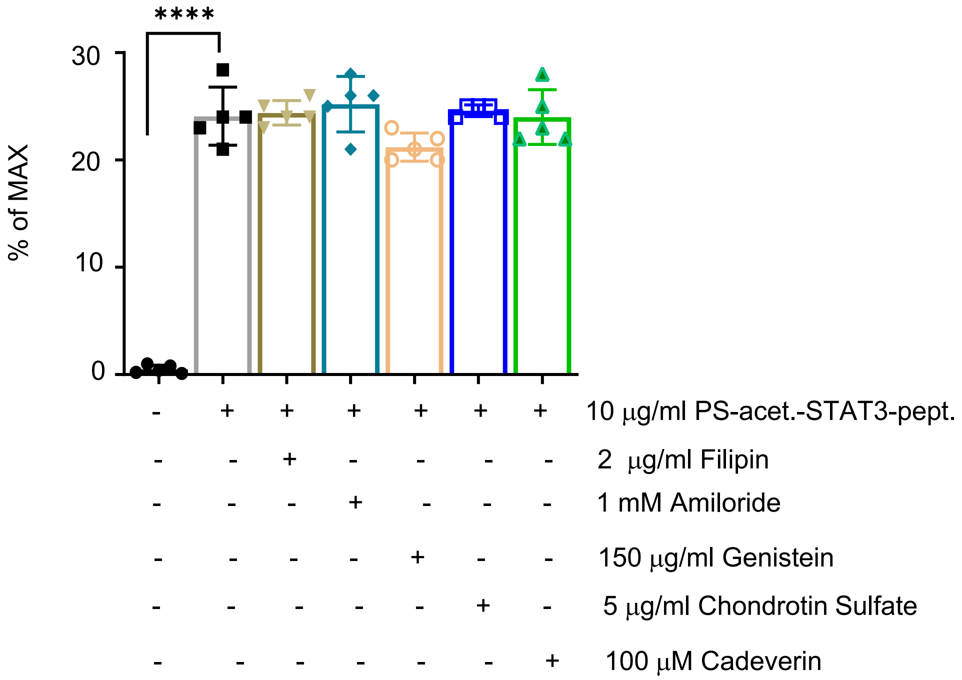
****
% of MAX
30
20
10
0
10 µg/ml PS-acet.-STAT3-pept.
2 µg/ml Filipin
1 mM Amiloride
150 µg/ml Genistein
5 µg/ml Chondrotin Sulfate
100 µM Cadeverin

C

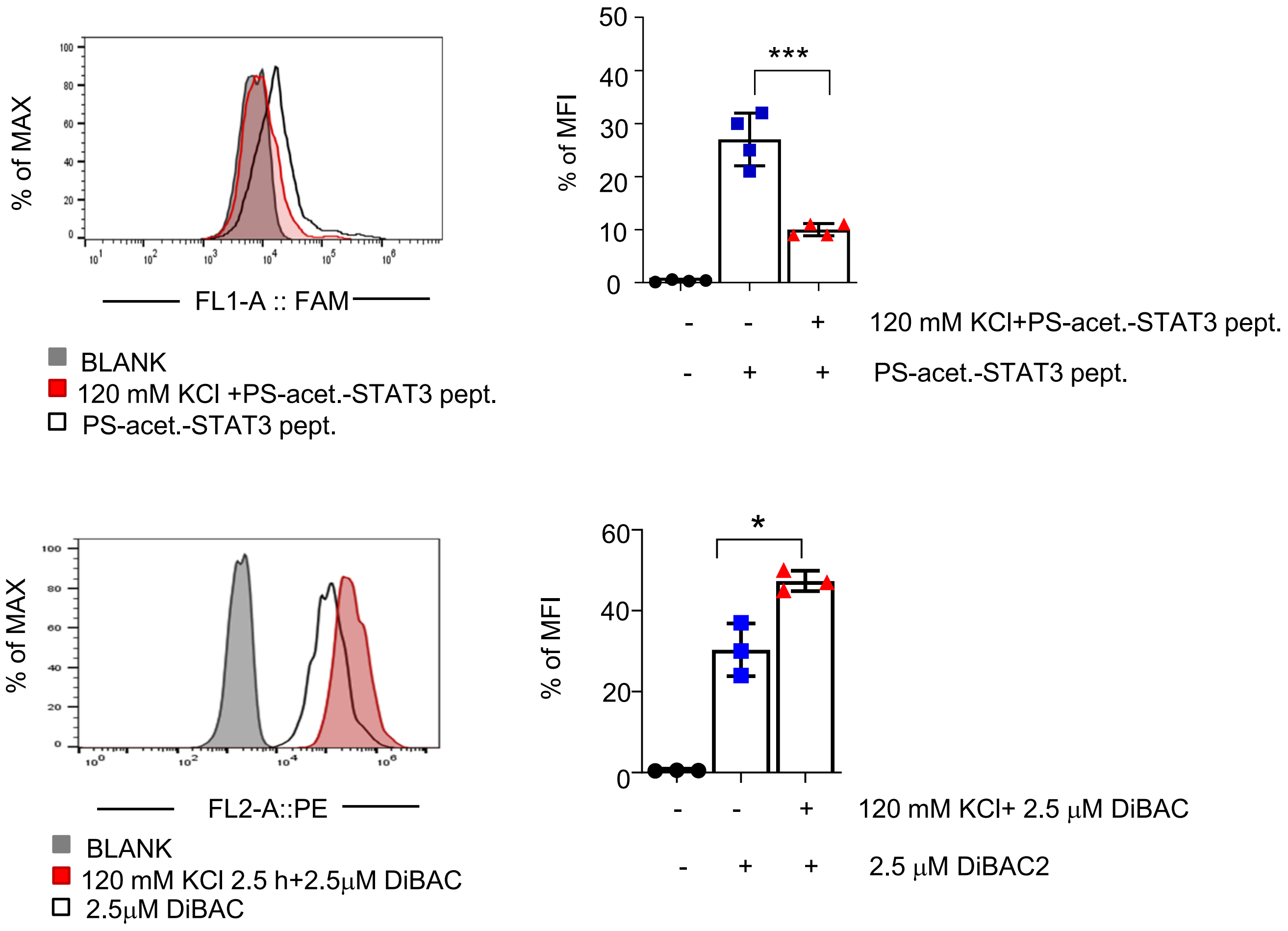
% of MAX
FL1-A :: FAM
BLANK
120 mM KCl +PS-acet.-STAT3 pept.
PS-acet.-STAT3 pept.
% of MFI
50
40
30
20
10
0
***
120 mM KCl+PS-acet.-STAT3 pept.
PS-acet.-STAT3 pept.
% of MAX
FL2-A::PE
BLANK
120 mM KCl 2.5 h+2.5µM DiBAC
2.5µM DiBAC
% of MFI
60
40
20
0
*
120 mM KCl+ 2.5 µM DiBAC
2.5 µM DiBAC2

Supplemental Figure 3 (related to Figure 1). The potential mechanism of PS-oligo mediated cell penetration.

(A) Dextran sulfate partially inhibits the peptide penetration into HCT116 cells. Peptide penetration was assessed by flow cytometry. SD is shown. Unpaired student *t*-test. **** P< 0.001. Data shown is the representative of 3 independent experiments.

(B) HCT116 cells were pre-treated with a variety of endocytosis inhibitors prior to treating with FAM-labeled PS-acet.-STAT3 peptide. The graph represents the statistical analysis of the fluorescence intensity (M.F.I) of FAM-labeled peptide shown in A. SD is shown. *n= 5* independent experiments. Unpaired student *t*-test. **** P< 0.001.

(C) Polarization of cell membrane contributes to penetration of PS-acet.-STAT3 peptide. Cell membrane was depolarized by adding 120 mM KCl to HCT116 for 2.5 h, followed by incubation with the peptide (upper panel). Data is representative of 4 independent experiments. Depolarization of the cell membrane was confirmed by applying 2.5 mM $DiBAC_4(3)$ to other cells (lower panel). *n= 3* independent experiments. Penetration was assessed by flow cytometry. The right panels show the mean of fluorescence intensity (M.F.I) quantified by FlowJo software. SD is shown. Unpaired student *t*-test. *, P<0.05; *** P< 0.005.

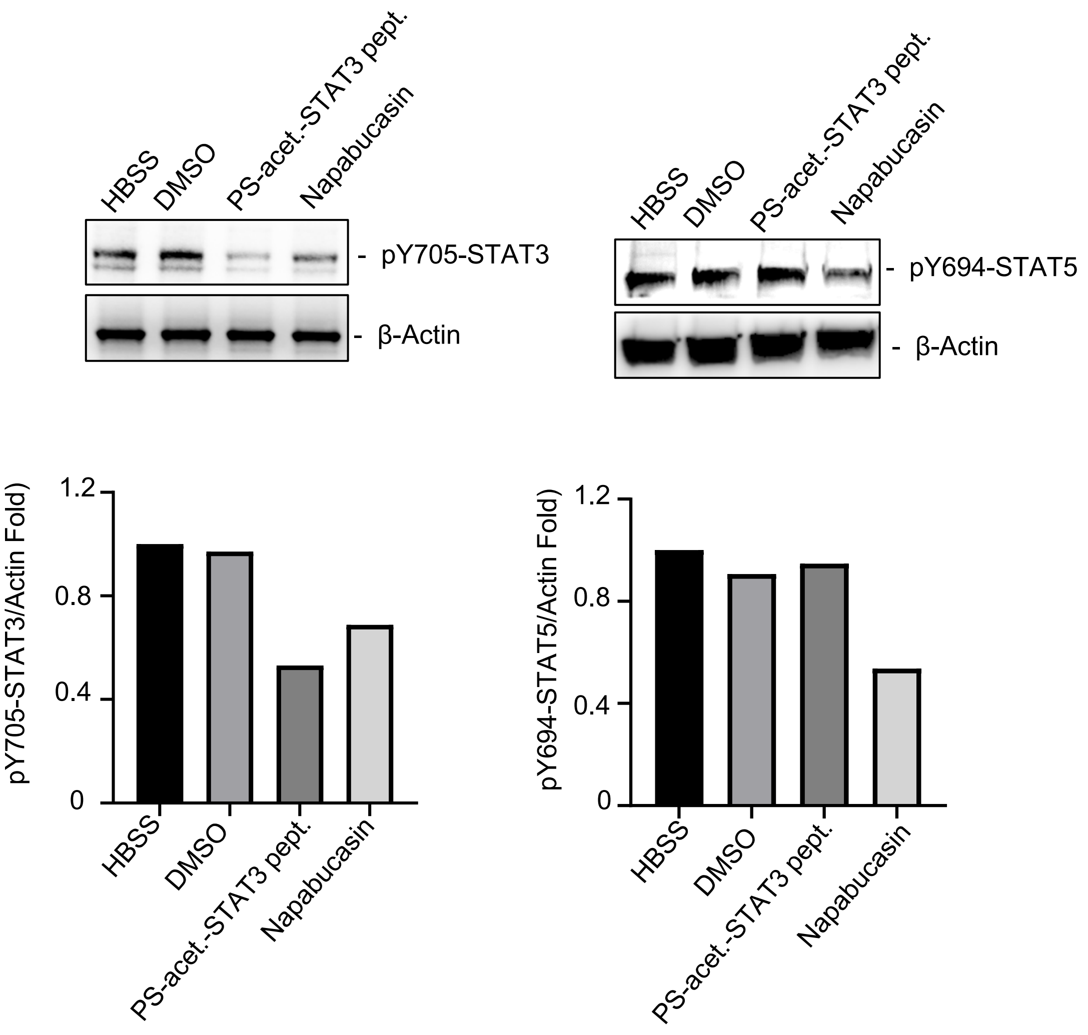

Supplemental Figure 4 (related to Figure 1). The target engagement of PS-acet.-STAT3 peptide. Phosphorylation of STAT3 at Y705 and STAT5 at Y694 was detected by Western blotting after 48 hours treatment of PS-acet.-STAT3 peptide (1 μM) or napabucasin (1 μM) in HCT116 cells.

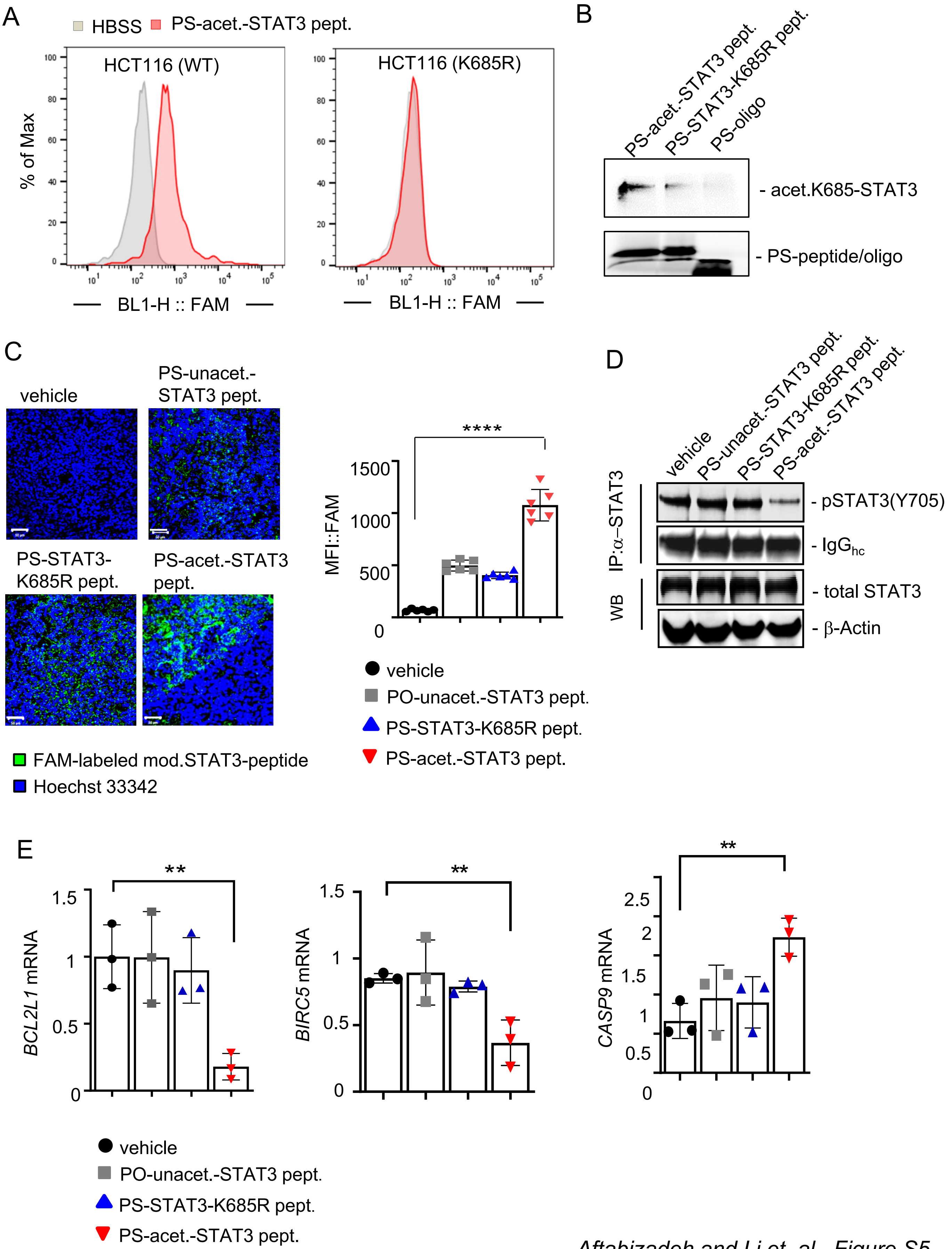

Aftabizadeh and Li et al., Figure S5

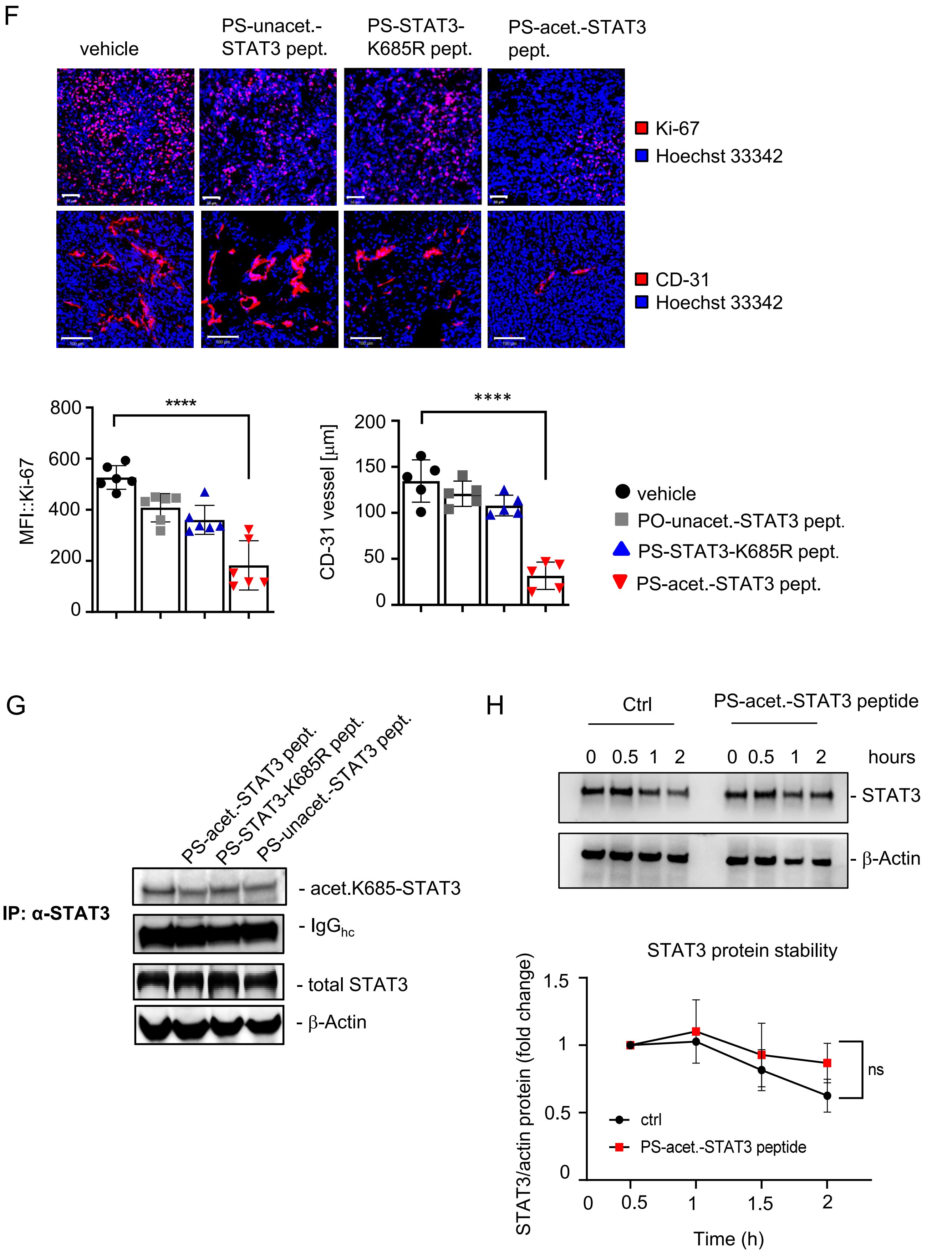

Aftabizadeh and Li et al., Figure S5

Supplemental Figure 5 (related to Figure 2). Cellular retention of PS-acet.-STAT3 peptide is enhanced in STAT3 wild-type HCT116 cells as well as tumors.

(A) K685R STAT3 mutant or STAT3 wild-type HCT116 expressing cells were treated with FAM-labeled PS-acet.-STAT3 peptide. The fluorescence intensity of FAM-labeled PS-acet.-STAT3 peptide in the cells was quantified by flow cytometry. Data shown is the representative of 3 independent experiments.

(B) PS-acet.-STAT3 but not PS-STAT3-K685R peptide specifically binds to acetyl-STAT3 in HCT116 cells which was demonstrated by immunoprecipitation of PS-oligo and Western blotting. PS-peptide or PS-oligo was visualized by SDS-PAGE and fluorescent gel imager.

(C to G) Subcutaneous HCT116 xenografts treated with FAM-labeled PS-unacet.-STAT3, PS-STAT3-K685R or PS-acet.-STAT3 peptides every other day at 1 mg/kg for 40 days.

(C) Retention of PS-acet.-STAT3 peptide was visualized with IF staining of tissue sections followed by confocal microscopy. *n= 6* tumor tissues. Scales, 50 μm. The right panel shows the quantification of FAM. One-way ANOVA; ****, $P<0.001$.

(D) STAT3 activation (pY705 phosphorylation) is suppressed by PS-acet.-STAT3 peptide treatment in HCT116 tumors that was proved by immunoprecipitation and Western blotting.

(E) The mRNA levels of *CASP9*, *BIRC5* and *BCL2L1* from the same tumor tissues described above were assessed by qRT-PCR. SD is shown. *n= 3* independent experiments. One-way ANOVA; **$P < 0.01$.

(F) IF staining of cell proliferation (Ki-67) and vascularization (CD-31) markers upon peptide treatments in HCT116 tumor tissues. Scales; 50 and 100 μm (upper panels). The lower panels show the quantification of Ki-67 and CD-31. *n= 6* tumor tissues. One-way ANOVA; ****, $P<0.001$.

(G) The level of acetyl-STAT3 was slightly decreased after PS-acet.-STAT3 peptide treatment in HCT116 tumors. Same protein samples were used from (D).

(H) The protein stability of STAT3 in HCT 116 cells was measured at different time points as indicated following cycloheximide (CHX) treatment. The cells were pre-incubated with 1 μM of PS-acet.-STAT3 pept. for 2 hours before adding 100 mg/ml of CHX. The protein level of STAT3 was detected by Western blotting. The quantification was analyzed by protein band intensity od STAT3 and actin by ImageJ software. *n= 3* independent experiments. SD is shown. Two-way ANOVA; ns, not significant.

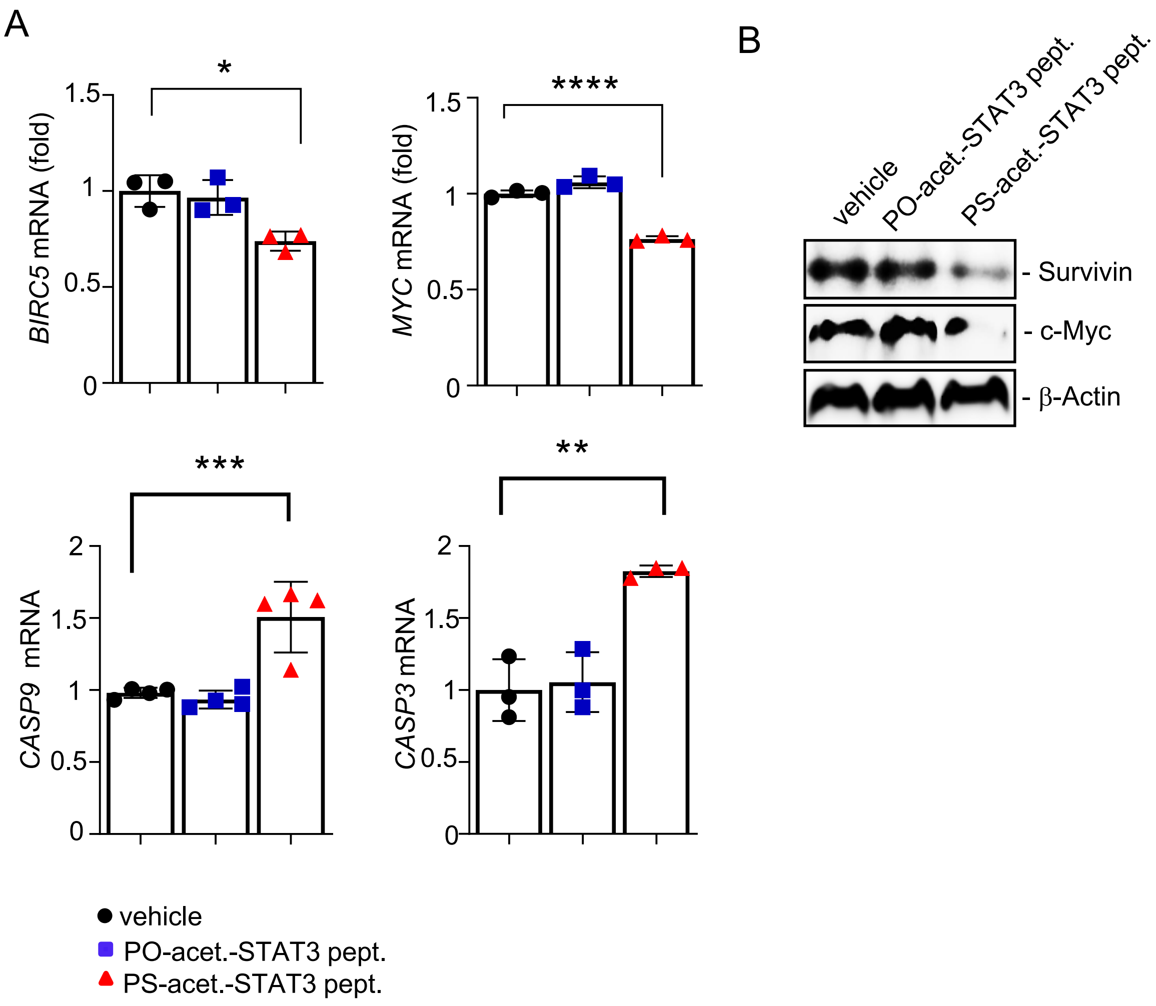

Supplemental Figure 6 (related to Figure 2). STAT3 inhibition regulated the expression of cell proliferation and apoptotic genes and proteins in human LoVo metastatic colorectal cancer cells. Protein and gene expression of STAT3 down-stream signaling after peptide treatment were assessed by (A) real time qPCR and (B) Western blotting. *n= 3* independent experiments. SD is shown. One-way ANOVA; ****; P<0.001; ***; P<0.005; **, P<0.01; *, P< 0.05

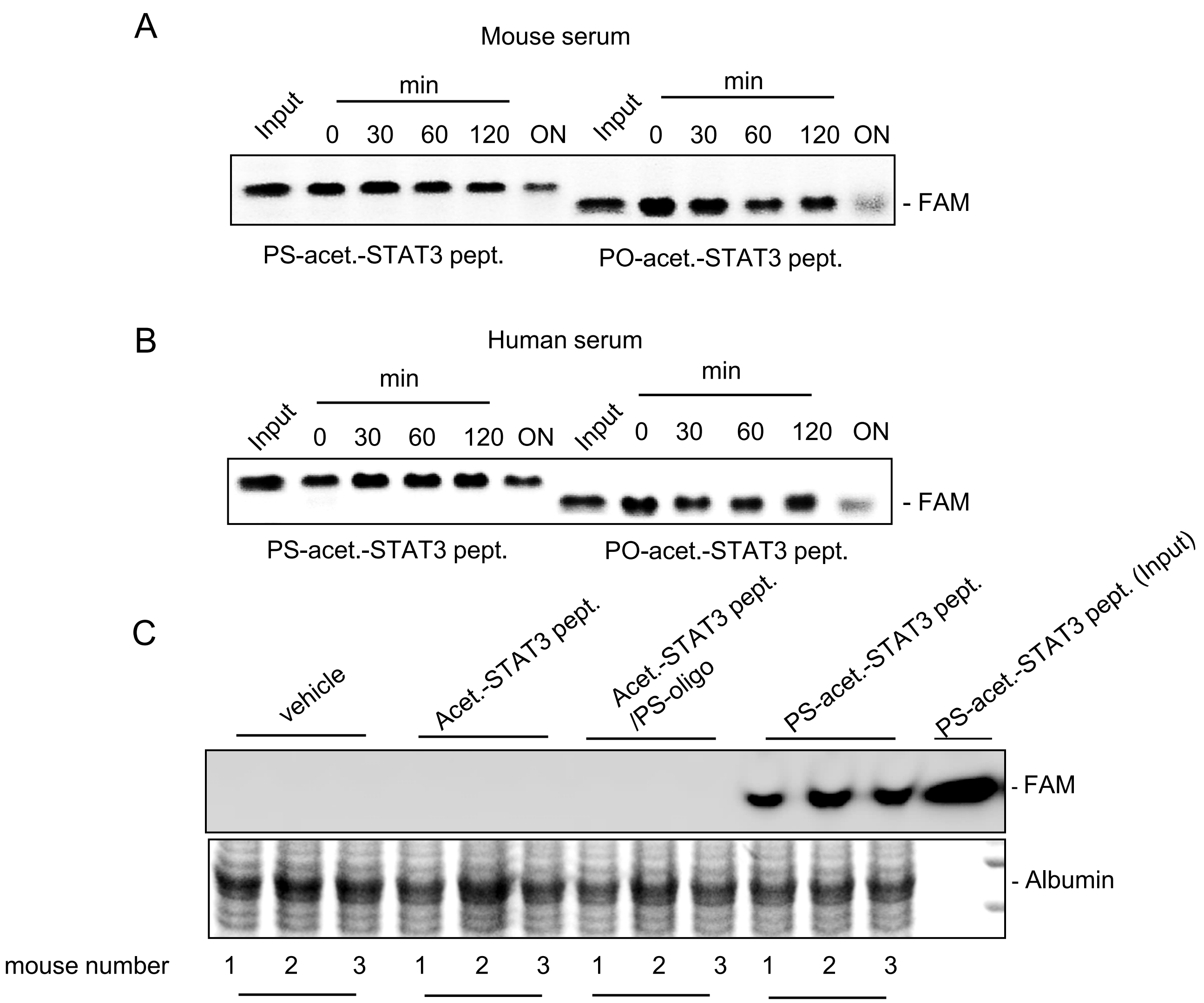

Supplemental Figure 7 (related to Figure 3). Conjugating phosphorothioated DNA oligonucleotides to acetyl-STAT3 peptide prolonged peptide's serum stability.

(A and B) Stability of FAM-labeled PS-acet-STAT3 peptide in (A) mouse or (B) human serum at the indicated time points was assessed by SDS-PAGE and visualized by a fluorescence gel scanner. A ChemiDoc imager (Bio-Rad) was used for imaging and analyzing fluorescence on the gels. ON, overnight.

(C) Stability of PS-acet.-STAT3 peptide in blood serum of NSG mice 24 hrs after treatment (*n=3*). The presence of FAM-labeled peptide in blood serum was assessed by SDS-PAGE (top). Serum albumin was stained with Coomassie brilliant blue. A ChemiDoc imager (Bio-Rad) was used to analyze the gel (bottom).

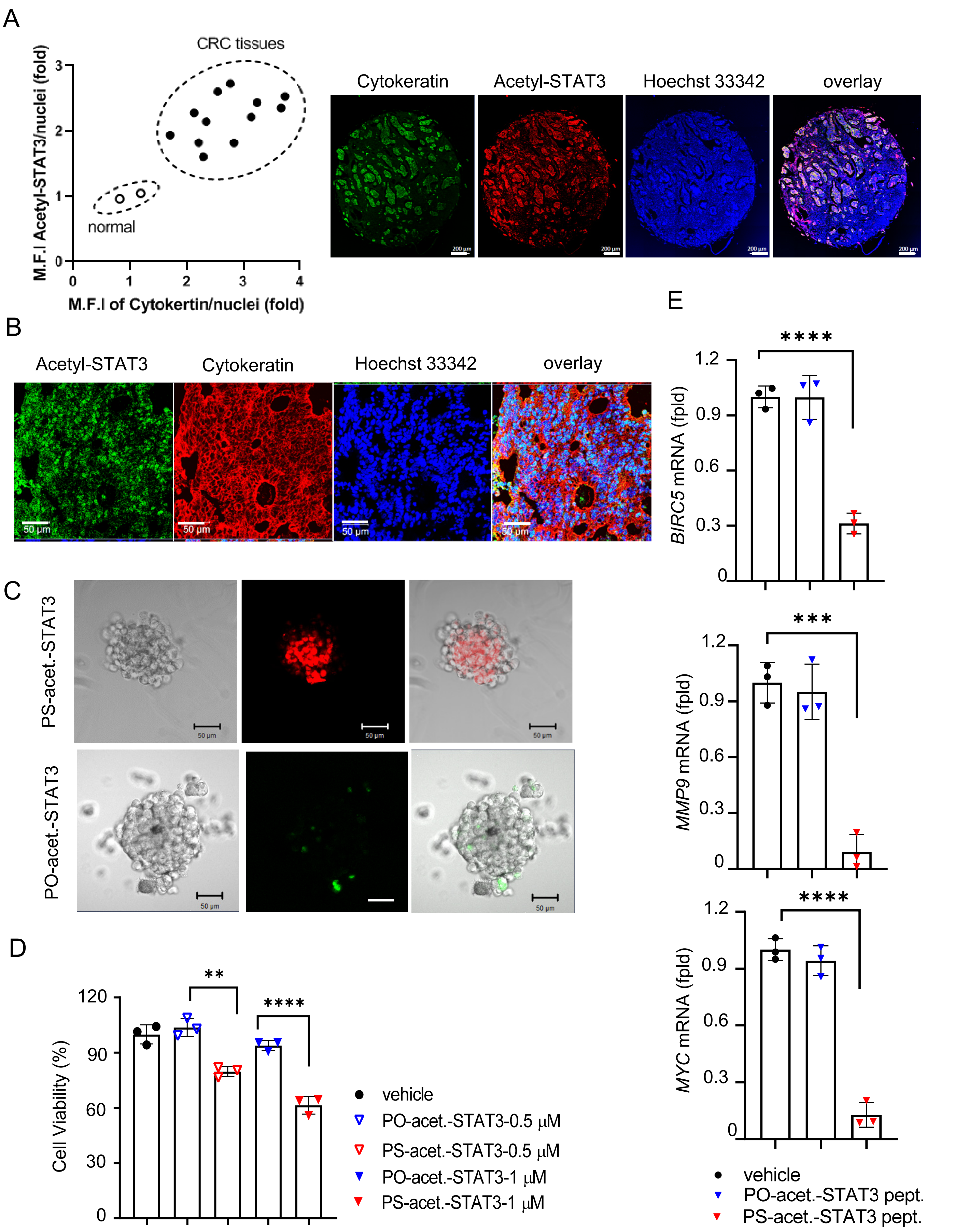

Aftabizadeh and Li et al., Figure S8

Supplemental Figure 8 (related to Figure 3). Acetyl-STAT3 protein is elevated in colorectal carcinoma (CRC) and PS-acet-STAT3 peptide treatment suppresses cell proliferation and STAT3 targeted gene expression of primary CRC tumor cells.

(A)The level of acetyl-STAT3 protein was examined in the tumor tissues from 12 CRC patients on a tissue array by IF staining. Images were examined by confocal microscopy and the mean of fluorescent intensity (M.F.I) was quantified by Zen software (Zeiss). Representative images are shown in the right panels. Scale bar, 200 μm. Cytokeratin was co-stained as a tumor cell marker. M.F.I of acetyl-STAT3 or cytokeratin was normalized with Hoechst 33342 in the plot.

(B) IHC staining of cytokeratin and acetyl-STAT3 in CRC PDX tumor sections. Scale bar: 50 μm.

(C) Highly efficient cell penetration of PS-acet.-STAT3 pept. in patient primary tumor sphere. PS-TAMRA-acet.-STAT3 (red fluorescence) and PO-FAM-acet.-STAT3 (green fluorescence) peptides were incubated with patient primary tumor sphere for 4 hours and the cell penetration was visualized by confocal microscopy. Scale bar: 50 μm.

(D) The primary CRC tumor cells derived from PDX were treated with different concentrations of PO- or PS-acet.-STAT3 peptide as indicated for 3 days. The cell viability was measured by CellTiter Glo assay and normalized with vehicle (HBSS) treatment. Data represent 3 independent experiments (*n=3*). SD is shown. Unpaired Student's *t*-test ****; P< 0.001; **, P< 0.01.

(E) Gene expression of *MYC*, *MMP9* and *BIRC5* was detected by qPCR and normalized with *ACTB* after vehicle, 1 μM of PO-or PS-acet.-STAT3 peptide treatment with CRC PDX tumor cells. Data represent 3 independent experiments (*n=3*). SD is shown. One-way ANOVA. ****; P< 0.001; ***, P< 0.005.

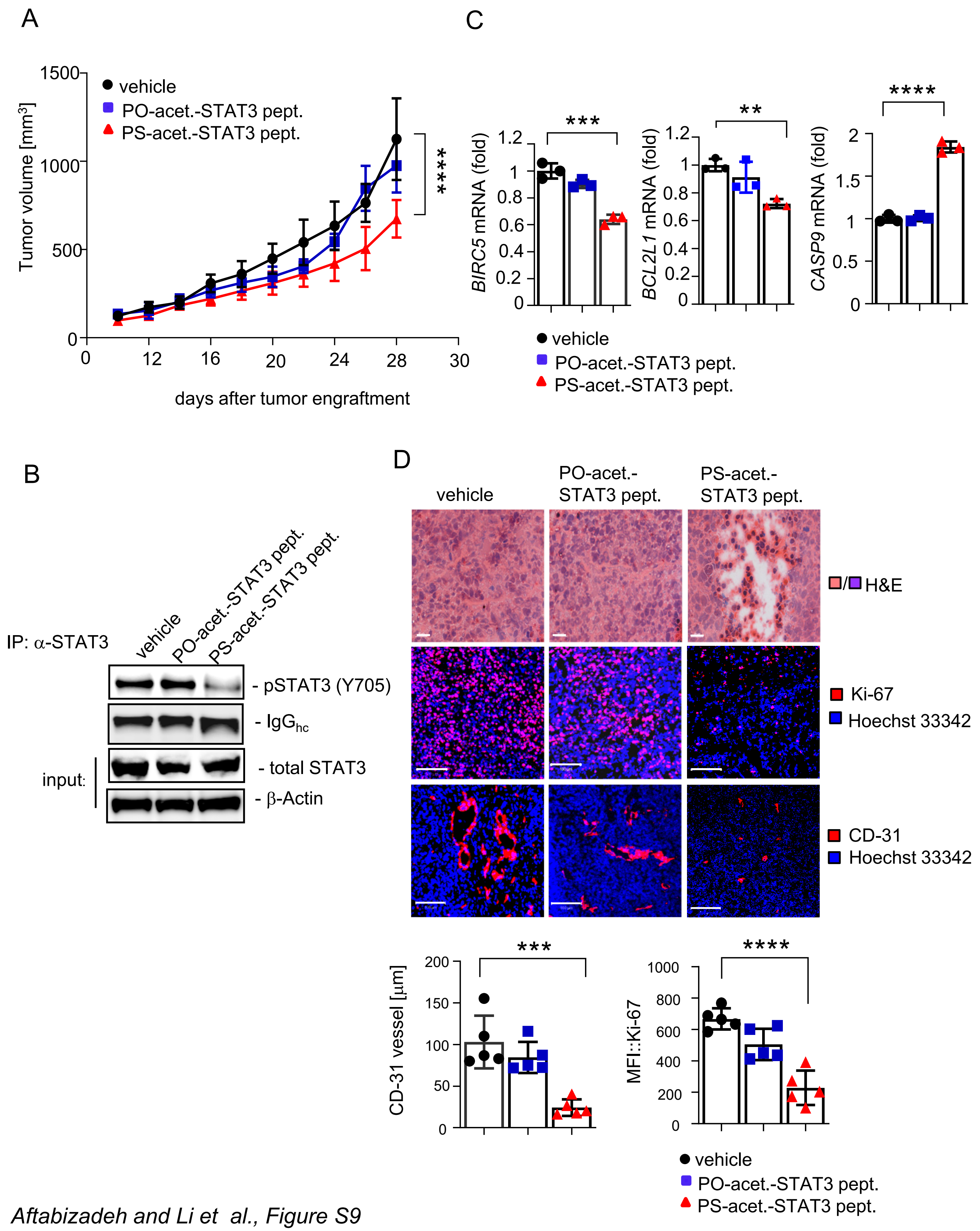

Aftabizadeh and Li et al., Figure S9

E

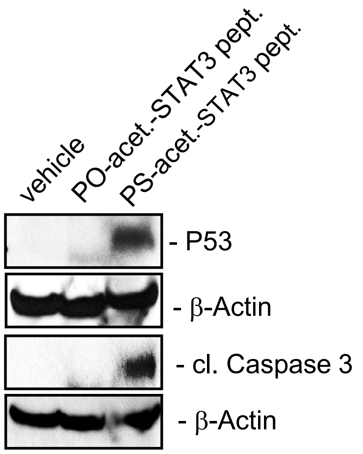
vehicle
PO-acet.-STAT3 pept.
PS-acet.-STAT3 pept.
- P53
- β-Actin
- cl. Caspase 3
- β-Actin

F

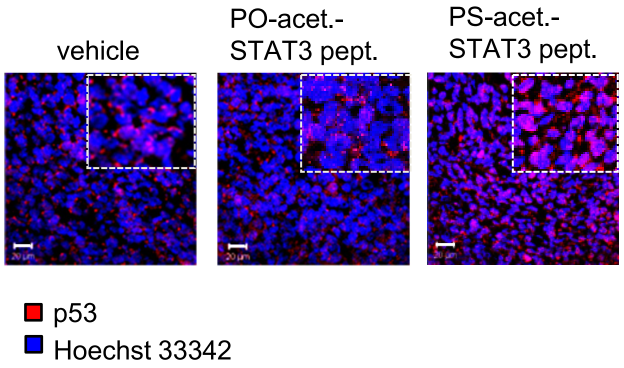
vehicle
PO-acet.-STAT3 pept.
PS-acet.-STAT3 pept.
p53
Hoechst 33342

G

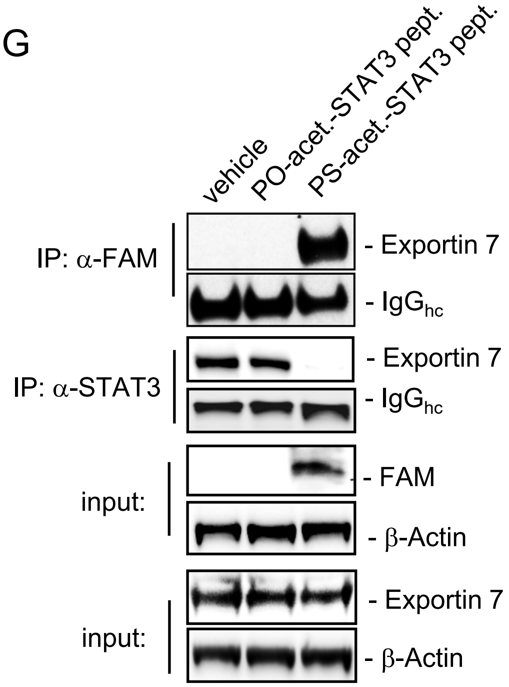
vehicle
PO-acet.-STAT3 pept.
PS-acet.-STAT3 pept.
IP: α-FAM
- Exportin 7
- IgGhc
IP: α-STAT3
- Exportin 7
- IgGhc
input:
- FAM
- β-Actin
input:
- Exportin 7
- β-Actin

Supplemental Figure 9 (related to Figure 3). The non-phosphorothioated (PO) acet.-STAT3 peptide does not show inhibitory effects on tumor growth.

(A) The growth kinetic of HCT116 tumors in NSG mice, treated locally with 1 mg/kg of PO- or PS-acet.-STAT3 peptides every day (*n=4*). SD is shown. Two-way ANOVA (Tukey's multiple-comparisons test) was used for analyze the kinetic of tumor growth over the treatment period; ****; P< 0.0001.

(B) PS-acet.-STAT3 peptide downregulates STAT3 phosphorylation, assessed by Western blotting using tumor homogenates prepared from tumors shown in A.

(C) mRNA levels for *CASP9*, *BIRC5* and *BCL2L1* in the same tumor tissues described above were assessed by qRT-PCR. SD is shown. One-way ANOVA. **, P< 0.01; ***; P< 0.005; ****P < 0.001.

(D) Treatments with PS-acet.-STAT3 peptide induced cell death, indicated by H&E staining (upper panel). Lower panels show Ki-67 and CD-31 protein expression levels. Panels show representative images from 4 tumors from each experimental group. Scale bars: 10 or 100 µm (data include 5 fields of view per group). Right panels represent quantification of Ki-67 protein levels and mean of vessel diameter (data include 5 fields of view per group). SD is shown. One-way ANOVA. ****; P< 0.001.

(E) Western blotting indicates the increased p53 and cleaved (cl.) Caspase 3 levels in the tumors shown in A.

(F) The increased p53 protein expression in HCT116 tumors treated with PS-acet.-STAT3 peptide is confirmed by IF followed by confocal microscopy imaging. The images represent tumor sections from 4 tumors per experimental group (*n=4*). Scales; 20 µm.

(G) Immunoprecipitation and Western blotting revealed PS-acet.-STAT3 peptide binds to exportin 7 protein and inhibits STAT3 binding to exportin 7.

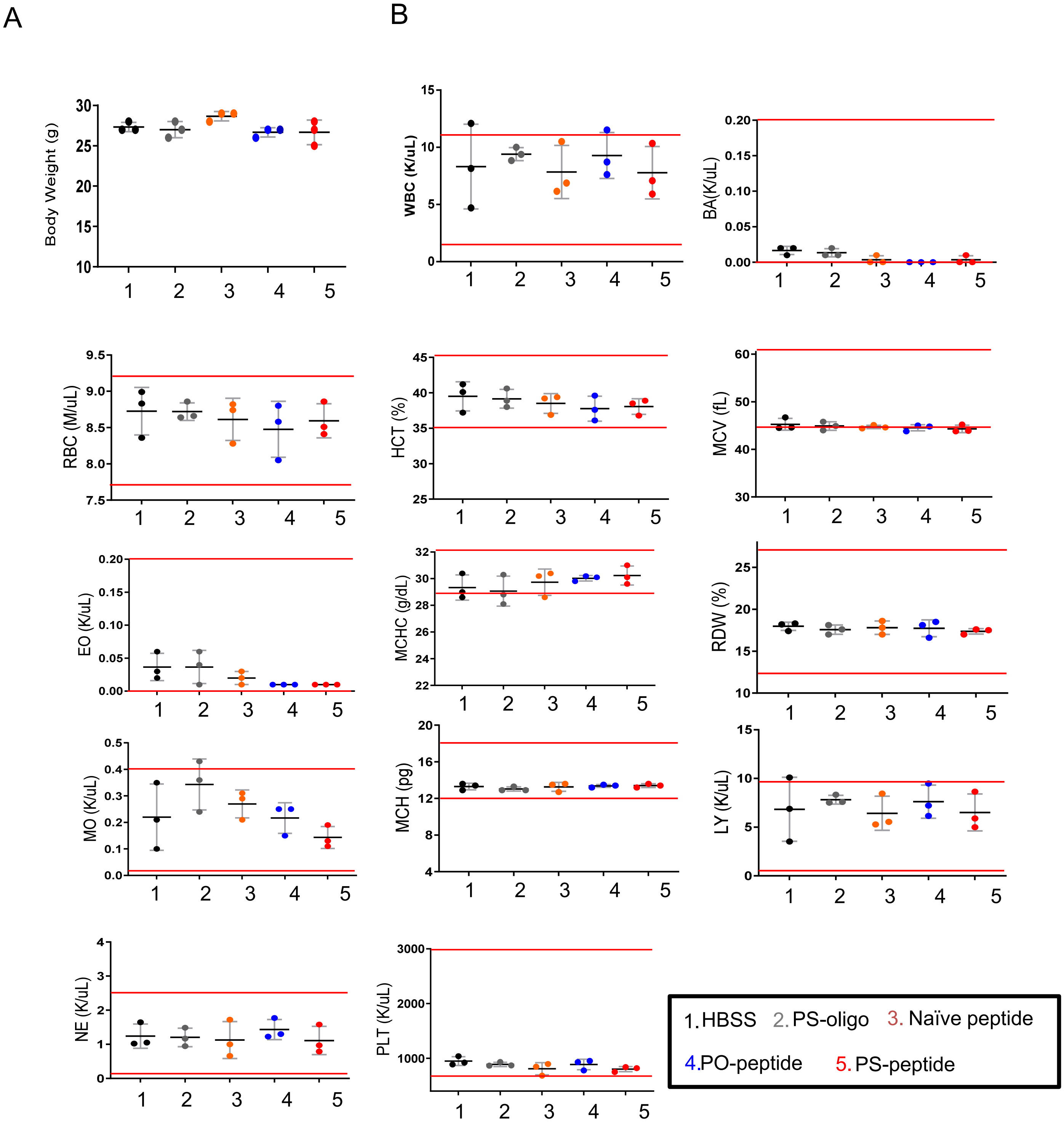

Aftabizadeh and Li et al., Figure S10

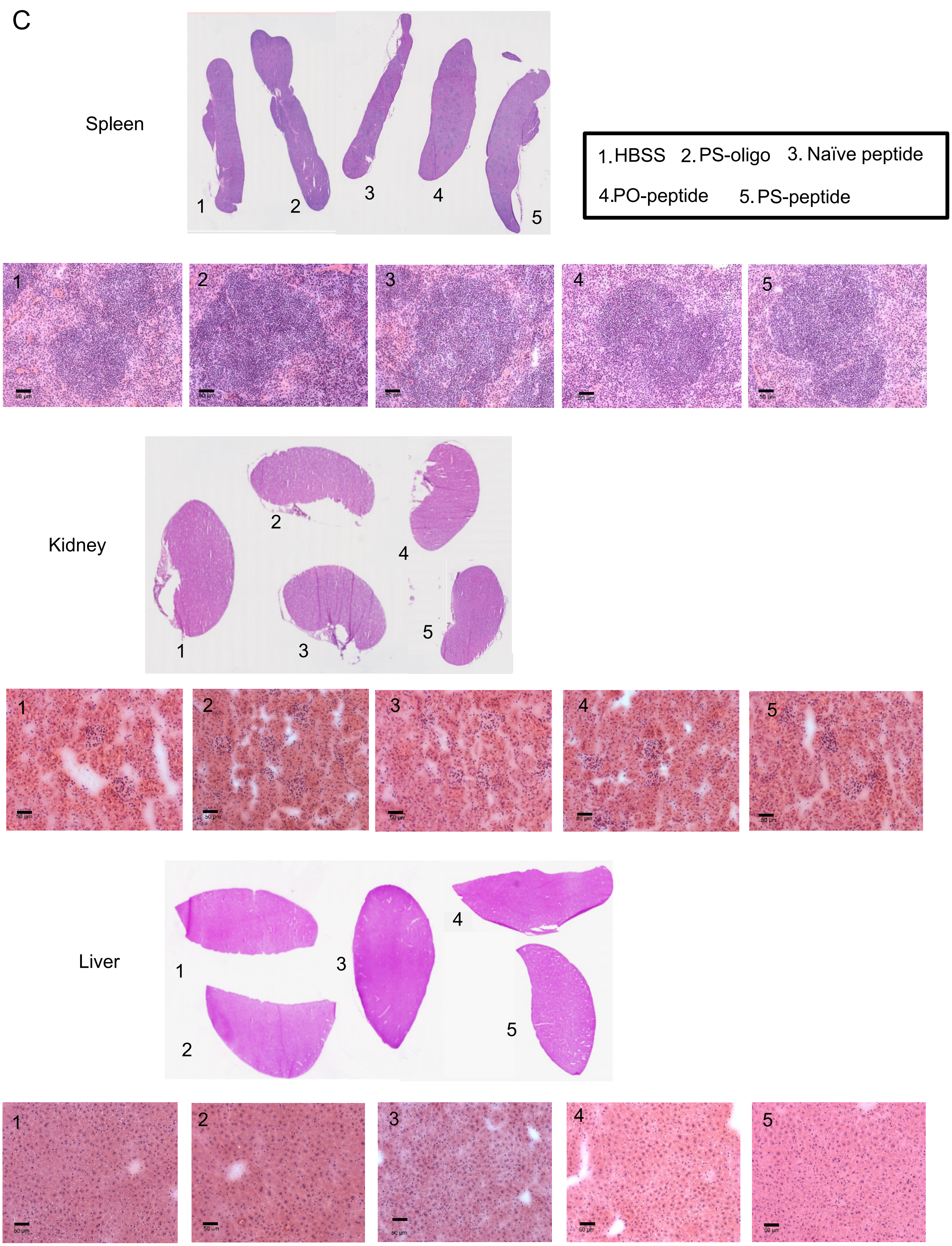

Aftabizadeh and Li et al., Figure S10

Supplemental Figure 10 (related to Figure 3). The systemic toxicity of PS-acet.-peptide. The bodyweight (A), blood count (B) and organ morphology (H&E staining; spleen, kidney and liver) (C) of the mice (*n=3*) treated with HBSS (1), PS-oligo (2), naïve peptide (3), PO-peptide (4) or PS-peptide (5) every other day at 1 mg/kg (per treatment) for 3 weeks. Individual value of each mouse is shown as a dot on the graph. The red lines on each graph in (B) indicate the normal ranges. Blood count was done by a veterinary hematology analyzer. The images shown in (C) are the represented H&E section from three mice (*n=3*). Enlarged view are shown below as indicated. Scale bar, 50 μm.

A

PS-acet.-STAT3 peptide

PDIPKEEAFGK685(Ac)YCRPESQEHPC-linker-T*C*C*A*T*G*A*G*C*T*T*C*C*T*G*A*T*G*C*T

PO-acet.-STAT3 peptide

PDIPKEEAFGK685(Ac)YCRPESQEHPC-linker-TCCATGAGCTTCCTGATGCT

PS-AS-acet.-STAT3 peptide

PDIPKEEAFG**K685(Ac)**YCRPESQEHPC-linker-A*G*C*A*T*C*A*G*G*A*A*G*C*T*C*A*T*G*G*A

PO-AS-acet.-STAT3 peptide

PDIPKEEAFG**K685(Ac)**YCRPESQEHPC-linker-AGCATCAGGAAGCTCATGGA

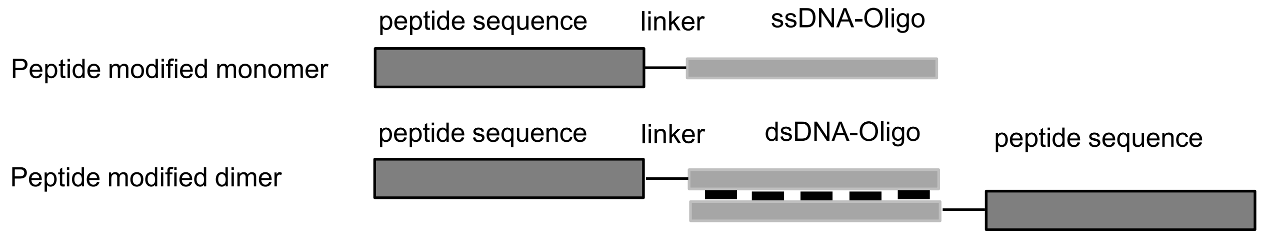

B

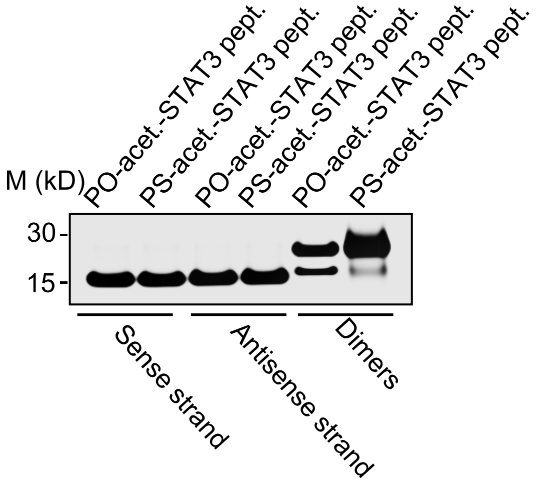

Supplemental Figure 11 (related to Figure 4). Oligo-based modification allows dimerization of the peptide.

(A) Complementary sequences of non-phosphorothioated (PO) and phosphorothioated (*, PS) DNA oligonucleotide used to dimerize the PO or PS-acet.-STAT3 peptide. Dimerization of the peptides is achieved by annealing anti-sense and sense DNA oligos.

(B) Dimerization of non-phosphorothioated (PO) and phosphorothioated (PS) DNA oligonucleotide-modified peptides is confirmed by running samples on a 4-12% gradient Bis-Tris gel using NuPAGE MES buffer.

Aftabizadeh and Li et al., Figure S11

A

PS-Gp130 peptide

MPKS(pY)LPQTV-linker-T*C*C*A*T*G*A*G*C*T*T*C*C*T*G*A*T*G*C*T

PS-MYC peptide

KNDTHQQDI-linker-T*C*C*A*T*G*A*G*C*T*T*C*C*T*G*A*T*G*C*T

PS-SC-MYC peptide

IDQNDTKHQ-linker-T*C*C*A*T*G*A*G*C*T*T*C*C*T*G*A*T*G*C*T

B

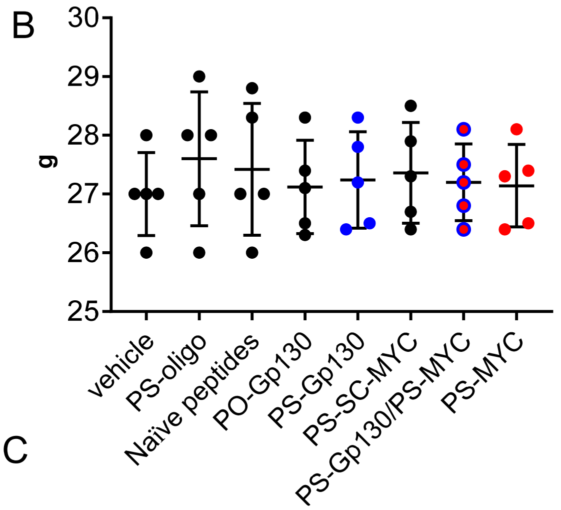

C

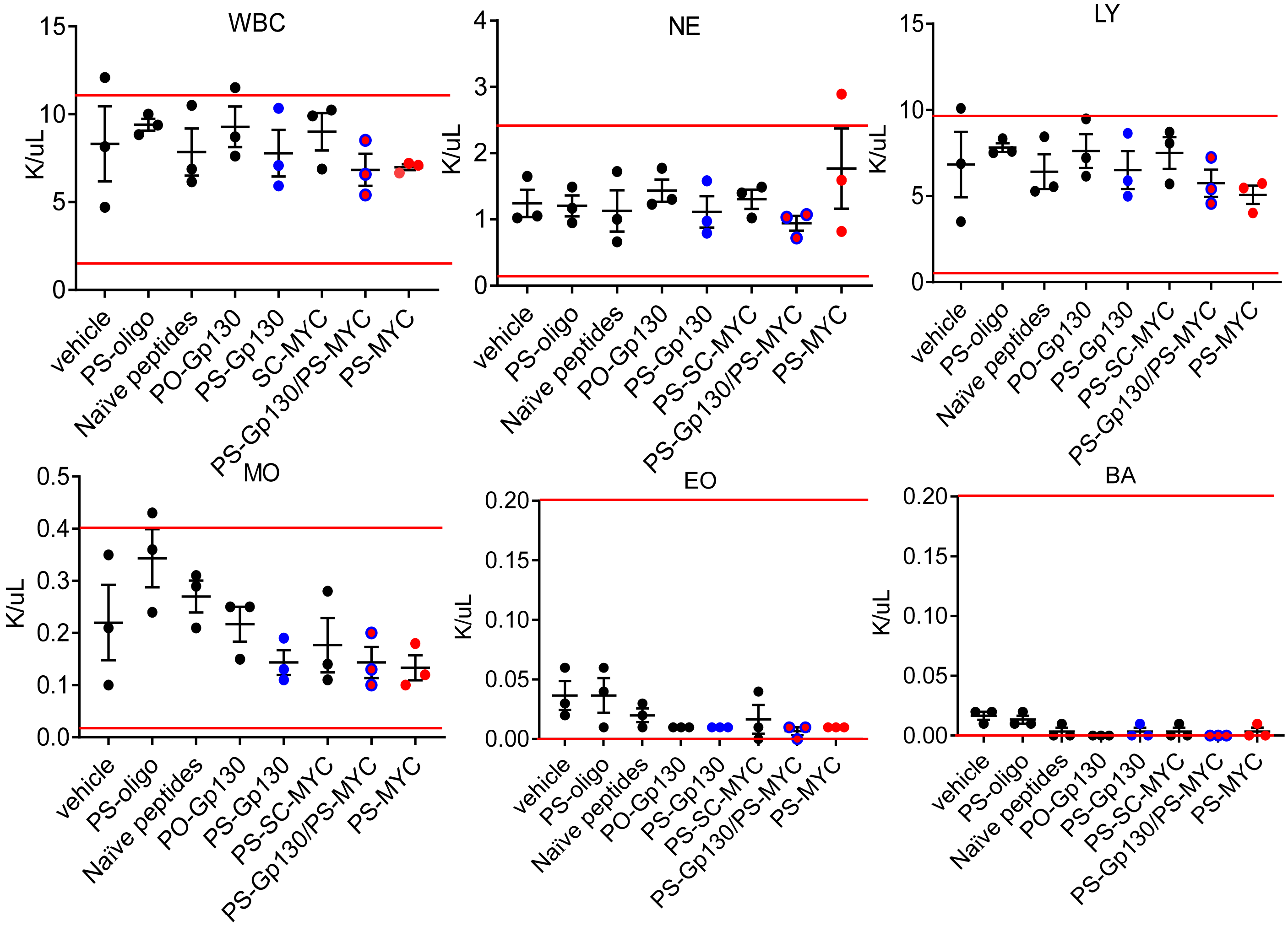

Aftabizadeh and Li et al., Figure S12

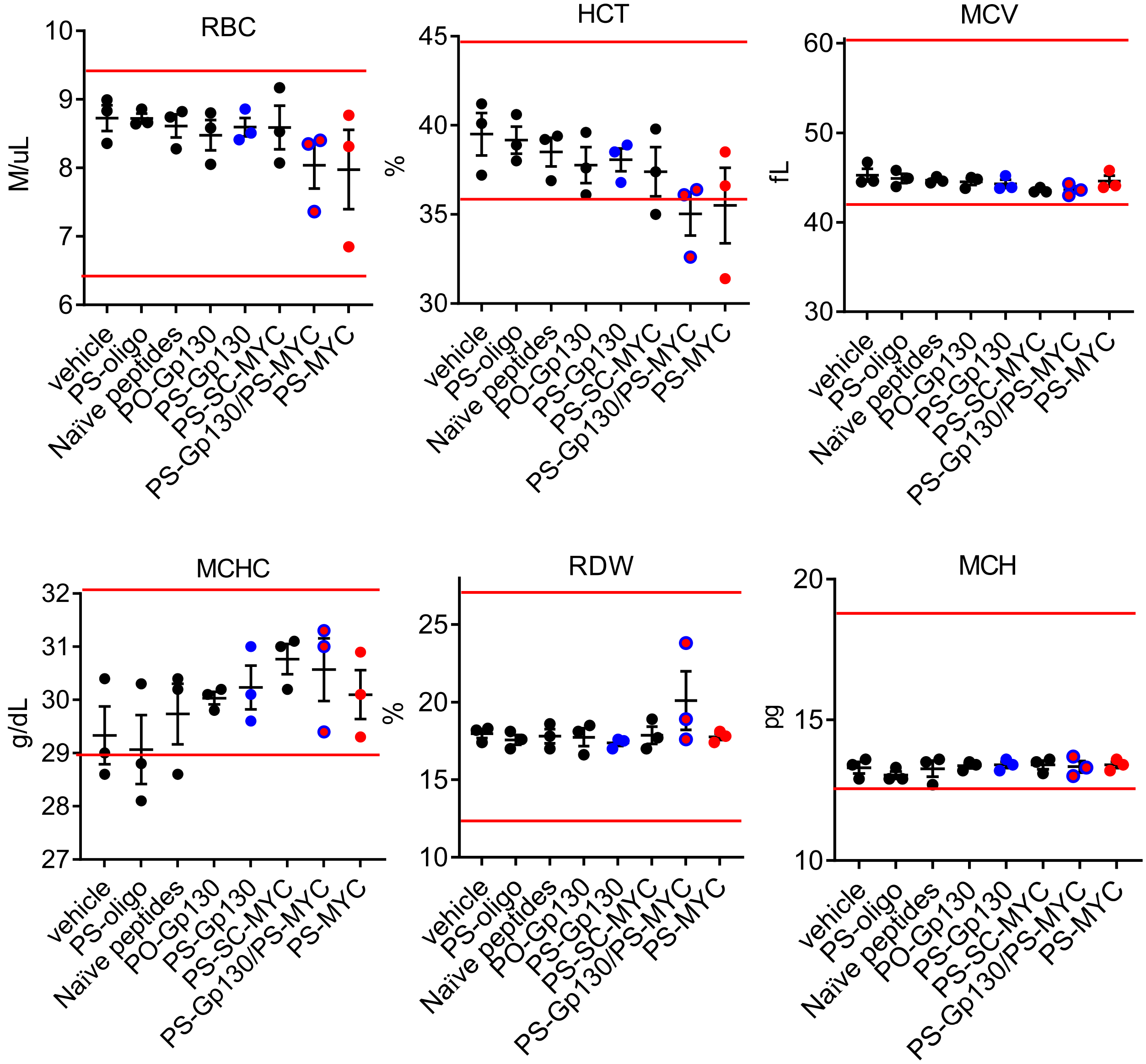

Aftabizadeh and Li et al., Figure S12

Supplemental Figure 12 (related to Figure 6).

(A) Amino acid sequences of phosphorothioated (*, PS) DNA oligonucleotide-modified Gp130 (PS-Gp130), MYC (PS-MYC) and scrambled MYC (PS-SC-MYC) peptides. PS-Gp130 peptide spans 9 amino acids of Gp130 protein (amino acids 901-910). PS-MYC peptide contains 9 amino acids of Max protein (amino acids 77-85).

(B and C) The bodyweight (B, *n=5*) and blood count (C, *n=3*) of the mice in each treatment group was measured at the end point. Individual value of each mouse is shown as a dot on the graph. The red lines on each graph indicate the normal ranges.
